# Unlocking the Potential of Hydrosols: Transforming Essential Oil Byproducts into Valuable Resources

**DOI:** 10.3390/molecules29194660

**Published:** 2024-09-30

**Authors:** Heloísa H. S. Almeida, Isabel P. Fernandes, Joana S. Amaral, Alírio E. Rodrigues, Maria-Filomena Barreiro

**Affiliations:** 1 Centro de Investigação de Montanha (CIMO), Instituto Politécnico de Bragança, Campus de Santa Apolónia, 5300-252 Bragança, Portugal; heloisa.almeida@ipb.pt (H.H.S.A.); ipmf@ipb.pt (I.P.F.); 2 Laboratório Associado para a Sustentabilidade em Regiões de Montanha (SusTEC), Instituto Politécnico de Bragança, Campus de Santa Apolónia, 5300-252 Bragança, Portugal; 3 Laboratory of Separation and Reaction Engineering-Laboratory of Catalysis and Materials (LSRE-LCM), Faculty of Engineering, University of Porto, Rua Dr. Roberto Frias, 4200-465 Porto, Portugal; arodrig@fe.up.pt; 4 Associate Laboratory in Chemical Engineering (ALiCE), Faculty of Engineering, University of Porto, Rua Dr. Roberto Frias, 4200-465 Porto, Portugal

**Keywords:** hydrosols, waste valorisation, bio-based circular economy, antimicrobial activity, antioxidant activity, bio-based ingredients and products

## Abstract

The global demand for sustainable and non-toxic alternatives across various industries is driving the exploration of naturally derived solutions. Hydrosols, also known as hydrolates, represent a promising yet underutilised byproduct of the extraction process of essential oils (EOs). These aqueous solutions contain a complex mixture of EO traces and water-soluble compounds and exhibit significant biological activity. To fully use these new solutions, it is necessary to understand how factors, such as distillation time and plant-to-water ratio, affect their chemical composition and biological activity. Such insights are crucial for the standardisation and quality control of hydrosols. Hydrosols have demonstrated noteworthy properties as natural antimicrobials, capable of preventing biofilm formation, and as antioxidants, mitigating oxidative stress. These characteristics position hydrosols as versatile ingredients for various applications, including biopesticides, preservatives, food additives, anti-browning agents, pharmaceutical antibiotics, cosmetic bioactives, and even anti-tumour agents in medical treatments. Understanding the underlying mechanisms of these activities is also essential for advancing their use. In this context, this review compiles and analyses the current literature on hydrosols’ chemical and biological properties, highlighting their potential applications and envisioning future research directions. These developments are consistent with a circular bio-based economy, where an industrial byproduct derived from biological sources is repurposed for new applications.

## 1. Introduction

Bioactive ingredients derived from medicinal and aromatic plants provide valuable ingredients for developing innovative pharmaceuticals, cosmetics, food products, and pesticide solutions. With the global rise of antimicrobial resistance, these plant-based compounds, e.g., essential oils and extracts, arise as environmentally friendly alternatives with antimicrobial properties. They hold significant potential for disease treatment and can be used in nutraceuticals, functional foods, and cosmetic formulations [1,2,3,4]. In this context, an emergent trend is the use of hydrosols, also known as hydrolates, byproducts of the distillation of aromatic plants to obtain EOs. These are complex mixtures containing traces of EOs but still exhibiting significant antimicrobial effects [5]. Therefore, although hydrosols are often discarded, strategies for residual EO recovery are being developed, such as liquid–liquid extraction or adsorption techniques. The recovered EOs, classified as secondary oils, can be blended with primary oils to correct organoleptic parameters. Although hydrosols can be used to recover EOs or pure compounds, these natural byproducts have attracted increasing scientific and technological interest as potential natural antimicrobial agents [6,7].

Aromatic plants have been used as food additives worldwide. In addition to improving organoleptic properties, they also increase a product’s shelf life. For this purpose, the early Egyptians used spices and EOs, which were also applied for centuries in China and India [8]. In the Iranian culture, hydrosols are used with sweeteners to prepare delicious natural drinks. In the Persian culture, depending on the selected plants, hydrosols are considered safe drinks and are often used for medicinal purposes; for example, the genus *Mentha* (Lamiaceae) is consumed to treat several diseases [9,10]. Hydrosols and alcoholic extracts of *Citrus aurantium* flowers are commonly used as food flavouring substances in a wide range of pastries and beverages across Cyprus and other Mediterranean regions, as well as to produce some anti-infectious, anti-depressant, sedative, and skincare agents for medical products [11]. In Turkey, hydrosols of medicinal plants are used for herbal drinks, acting as natural antibiotics against bacterial diseases and aiding digestion [12]. Additionally, hydrosols can also be applied to prevent biofilm formation and can be used as sanitising agents in food and medical environments to enhance food quality and reduce safety risks [13,14]. Several common hydrosols are sold globally and used commercially (such as rose, lavender, peppermint, and lemon), with their popularity rising, especially in aromatherapy. Moreover, herbal and floral hydrosols are now commercially available; for example, in Italy, they are used in cosmetic formulations or sold in their pure form for food applications [15].

Due to their interest and versatility, these byproducts can be a sustainable approach for different technological applications, according to the circular economy’s principles, facing the environmental threats posed by residue generation [16]. Besides hydrosols, the solid biomass residue of the EOs extraction can also be explored to generate economic value. In this regard, Zaccardelli et al. [17] have studied the oil-free biomass from basil, rosemary and sage, proposing their reuse as composting. They concluded that these residues could be successfully applied to restore and maintain soil fertility. Alternatively, Turrini et al. [18] investigated using lavender biomass as a source of bioactive (polyphenols, particularly flavonoids), demonstrating the interest in the generated compounds for the agronomic, food, and cosmetic areas.

Considering sustainability concepts, when discussing hydrosols, a double challenge must be considered: waste reduction and safety. In this context, the primary aim of this work is to compile and present information on the byproducts of essential oil extraction, with a particular focus on hydrosols and their potential for sustainable applications. The various sections of this work provide an overview of the characteristics of these biowastes, emphasising their obtainment methods, chemical and biological properties, and applications. The gathered studies have pointed out the need to standardize these products, fostering research into consumer safety and the development of eco-friendly products. Future perspectives outlined in this review underscore hydrosols as promising candidates to use as natural antimicrobial agents.

## 2. Bibliometric Analysis

This section collects, analyses, and summarises the existing literature on hydrosols as potential natural antimicrobials. Scientific search engines, such as ScienceDirect, SpringerLink, Web of Science, Scopus, Wiley Online Library, and Google Scholar, were used to collect the published literature. Several keywords were used for the search, namely combining “hydrosols” and “hydrolates” with “biological activities”, “bioactivity”, “antimicrobial activity”, “antioxidant activity”, “antifungal activity”, “chemical characterisation”, “chemical composition”, “food applications”, “food preservatives”, “hydro distillation”, “steam distillation” and “distillation process of aromatic plants.” The analysed references correspond with the retrieved papers, which were also examined to identify additional relevant works.

**Figure 1:**
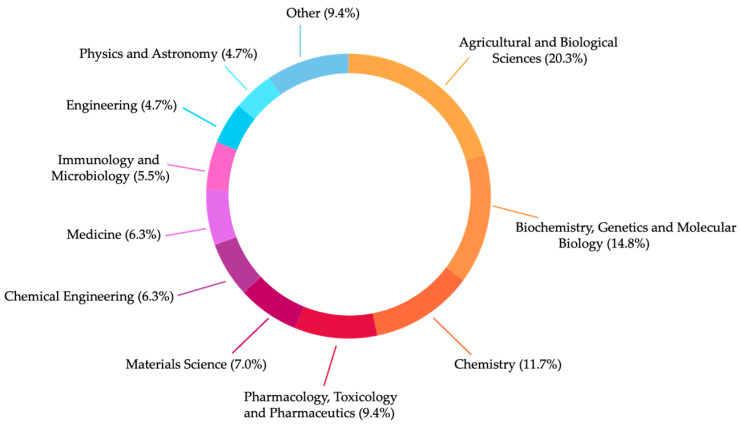
Published documents from 2014 to 2024 about hydrosols and antimicrobial activity, categorised by subject area.

Figure 1 summarises, by subject area, the descriptive analysis of the gathered data from 2014 to 2024 using “hydrosols”, “antimicrobials”, and “activity” in the Scopus database. This highlights the importance of valorising and applying this byproduct across various fields.

**Figure 2:**
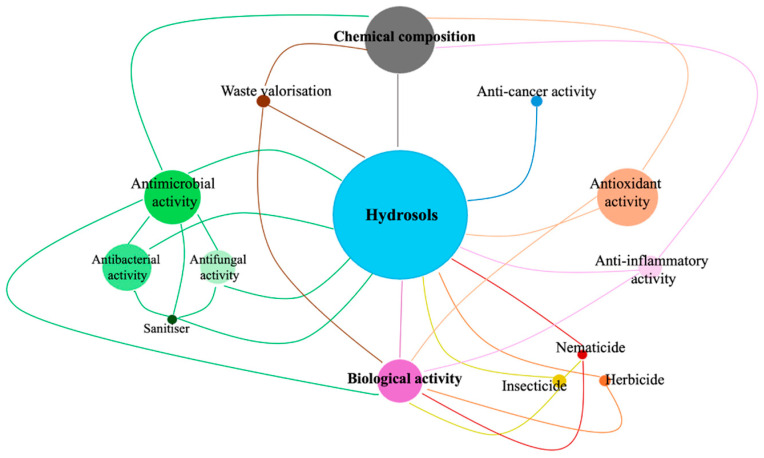
Graph clustering of published documents on the most relevant topics related to hydrosol characterisation, with groups and their interconnections depicted in different colours.

Analysing the published documents allows for the identification of the most frequently addressed topics in hydrosol research. This is illustrated in Figure 2, where the size of each circle is proportional to the number of published works. The descriptive analysis, which compiled data from the Scopus database spanning 2014 to 2024, reveals that the most extensively researched topics are “biological activity” and “chemical composition.” These areas are closely linked to hydrosol characterisation, with antioxidant and antimicrobial properties being the most studied bioactivities. Moreover, recent trends in the literature emphasise emerging topics, including anti-cancer and anti-inflammatory bioactivities, hydrosols as biopesticides (insecticidal, nematocidal, and herbicidal applications), and waste valorisation.

## 3. Hydrosols

### 3.1. Distillation Processes

Different methods can be used to obtain EOs, including water distillation-based techniques, cold pressing, supercritical CO_2_ extraction, Soxhlet extraction, and some emerging techniques, such as microwave- and ultrasonic-assisted extractions. The objective is to isolate volatile compounds from plants, which are present in lower quantities but offer significant added value. Water distillation is the traditional method for EO production, accounting for 93% of the total EO production. Two approaches can be employed based on how the water interacts with the aromatic plants during extraction: hydro distillation (HD) or steam distillation (SD). In this process, two immiscible phases are formed during condensation, where the “hydrosol” is the aqueous phase byproduct [11,19,20,21,22]. Hydrosols contain EO traces (usually less than 1 g/L) and other water-soluble plant bioactive compounds. They are traditionally characterised by their dilute nature and high acidity (pH ranging from 3.5 to 6.5). They have a herbal aroma that changes from mild to intense and from pleasant to unpleasant, which can be, in some cases, similar to the extracted EO, depending on the collection phase during distillation. This byproduct assumes different labels in the literature: hydrosols, hydrolates, hydroflorates, aromatic plant waste, aromatic water, floral water, and essential aromatic water [12,14,23,24]. The byproduct is easily obtained and must be stored in aseptic containers, sealed and maintained in a cool place.

**Figure 3:**
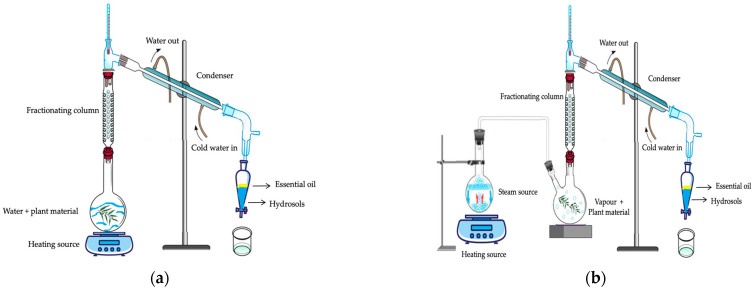
Schematic representation of (**a**) hydro distillation and (**b**) steam distillation setups.

Aromatic plant distillation can be undertaken from different plant parts, such as flowers, fruits, roots, leaves, bark, and resins. During the process, the vaporised EO remains, for an extended time, in contact with a large volume of steam and/or condensed water. As a result, the EO phase contains most of the volatile compounds. In contrast, the hydrosol phase includes polar, oxygenated, odour-contributing, hydrophilic and trace amounts of volatile oil compounds. These components tend to preferentially partition into the hydrosol phase due to their ability to form hydrogen bonds with water [11,25,26,27]. Considering the conditions of the steam distillation method, the plant material remains in contact only with steam. In hydro distillation, the plant material interacts directly with the water phase. When plants are exposed to steam or boiling water, they produce a mixture of vaporised water and oil. This mixture is then condensed, with the essential oil and hydrosol separated based on their density differences and collected, as illustrated schematically in Figure 3. Overall, different products are obtained in the extraction process, namely, EO, hydrosol, solid residue (biomass) for both HD and SD and decoction water (for HD) [28].

Steam systems are preferred on an industrial scale over hydro distillation because they can process larger quantities of raw materials, reducing investment costs and minimising the hydrolytic degradation of compounds. Using vapour from boiling water lowers process temperature by decreasing the partial pressure of the components in the vapour phase, which helps prevent decomposition, especially when processing matrices with low-volatility compounds. In hydro distillation systems, prolonged exposure of plant material to boiling water can lead to the hydrolysis of esters, polymerisation of aldehydes, and degradation in general. There are also green alternatives, such as microwave extraction, which uses microwave radiation to heat the water, reducing the required amount and the operation time. This method is primarily designed for hydro distillation but can also be adapted for steam distillation [19,29]. At the laboratory scale, hydro distillation is frequently employed because it requires less water and is suitable for small-scale operations. Typically, a Clevenger apparatus or a modified distillation setup is used.

Official pharmacopoeias define EOs as a complex, fragrant product that can be extracted with different distillation systems. Nevertheless, the absence of legal definitions, specifications, or standards for their use highlights the importance of further research. More studies are needed regarding the scientific validation of hydrosols’ chemical and biological composition [15,16,27]. Standardising these products will ensure quality and safety, paving the way for various potential applications. Documenting and scientifically evaluating hydrosols could be an important step towards product authentication, contributing to expanding the range of available natural additives for industrial applications [9]. Moreover, standardised extraction procedures are crucial to prevent nomenclature issues with essential oil extraction byproducts, especially to ensure that wastewater or decoction residues are not mistakenly labelled as hydrosols [30,31,32,33,34].

### 3.2. Chemical Characterisation

The chemical characterisation of EOs and hydrosols is crucial for understanding their composition and assessing their potential. Chemotype characterisation is crucial for understanding hydrosols’ bioactivity. Safety can be preliminarily assessed by analysing their composition compared with their corresponding essential oils and the existing literature. For example, carvacrol, the main component in the EOs of oregano and thyme is also found in their hydrosols. Carvacrol is a potent antimicrobial agent with strong antioxidant properties and is effective against fungi and foodborne pathogens. It is classified as a generally recognized as safe (GRAS) substance by the Food and Drug Administration (FDA). It is registered by the European Commission as safe for consumers, with a maximum survey-derived daily intake of 0.0003 mg/kg body weight per day and a no-observed effect level of 500 mg/kg body weight per day [35,36].

The quality of hydrosols is determined by the concentration of their soluble volatile compounds, which, for a given plant species, is influenced by various factors, including environmental conditions (e.g., temperature, rainfall, day length), harvesting conditions (e.g., season, geographical location, growth stage, time of day), and post-processing of the plant material (e.g., drying) [6,37]. In addition to the variability of the raw material, distinct distillation processes may also affect the hydrosol composition. Moreover, screening studies have revealed that the sample collection time during distillation may jeopardise their chemical composition and odour [9]. The vast diversity and abundance of compounds make their analysis challenging, with gas chromatography coupled with mass spectrometry (GC–MS) being the most commonly used technique [38]. Before GC–MS analysis, liquid–liquid extraction (LLE), employing solvents with varying polarities, can be employed to extract the compounds from the hydrosols. Kokalj Ladan et al. [39] investigated different pathways for studying hydrosols’ chemical compositions using solvent extraction and direct analysis. This study has indicated that direct analysis is more appropriate for detecting adulteration with hydrophilic compounds. The solvent extraction method (e.g., LLE) is more suitable for detecting compounds present at low concentrations. In the work of Ndiaye et al. [40], hydrosols of different *Eucalyptus* species, obtained after a 2 h distillation, were subjected to LLE with n-hexane. In the study of Ghavidel et al. [41], where different hydrosols (4 h distillation) were characterised, petroleum ether was used for the LLE. Both authors have identified the major hydrosols’ components. Two approaches that do not require a prior extraction step are headspace solid-phase microextraction (HS-SPME) and purge-and-trap automatic thermal desorption (P&T-ATD) [41,42].

Several studies have reported the chemical characterisation of hydrosols obtained from various plants, focusing on the most common extraction techniques: hydro distillation and steam distillation. Regarding hydro distillation, Benoudjit et al. [43] studied the *Cymbopogon citratus* hydrosol, detailing its organoleptic, physicochemical, and structural characteristics through Fourier transform infrared spectroscopy (FTIR). The analysis revealed that the hydrosol is a clear liquid with a pH of 6.0, a relative density of 0.998 and a refractive index of 1.333 (20 °C). It has a fresh, strong lemon scent and contains functional groups such as alcohols, alkanes, aldehydes, alkenes, and aromatic rings. Selles, Lemouchi and Did [44] characterised *Teucrium kabylicum* Batt., showing that the EO chemical composition is mainly based on monoterpene hydrocarbons and aliphatic compounds, and that the hydrosol is primarily composed of oxygenated components like limonene, 1,2-epoxide, citronellal, and terpinene-4-ol, presenting a noteworthy array of bioactive compounds. Lei et al. [45], who investigated eight *Paeonia suffruticosa* Andr. cultivars from central China, also reported chemical differences between the EOs and the hydrosols. Due to the predominance of oxygenated compounds and trace levels of hydrocarbons, they have concluded that *Paeonia suffruticosa* hydrosol could be a viable alternative to EOs. Regarding the variability of hydrosols, Grubešić et al. [46] examined the phytochemical profile of essential oils and hydrosols from *Olea europaea* L. cv. Oblica leaves, reporting a consistent chemical composition across the different vegetative phases over six months. The major compounds included α-pinene, β-ionone, myristicin, docosane, 1-hexanol, oleic acid, and (E)-β-damascenone. These compounds are linked to relevant biological activities, showing their potential for food preservation or cosmetic and pharmaceutical ingredients. Dobreva et al. [47] compared the yield and chemical composition of rose EOs and hydrosols from industrial plantations in Bulgaria. The results suggest that variations in the quality of essential oils and hydrosols are associated with plantations of different *Rosa damascena* genotypes, including unidentified rose varieties. These differences may also be influenced by geographical factors, climatic conditions, and processing methods. Variations in organoleptic properties and bioactivities can potentiate different applications. Shiati et al. [48] evaluated the chemical composition of *Echium amoenum* hydrosols used in Iran as medicinal drinks. They compared their quality with that of a standard hydrosol produced in the laboratory, both extracted by HD. Differences were noticed, and/or impurities were identified in commercial products. In another study [49], *Torreya grandis* hydrosols were subjected to different treatments (membrane filtration and homogenisation), revealing significant changes in the composition between the filtered hydrosol and the non-homogenised and homogenised versions. Though both treatments enhanced hydrosol stability, the hydrosol subjected to high-pressure homogenisation was the only one demonstrating a significant increase in bioactivity (e.g., antioxidant activity). Lahmar et al. [50] identified enzymes in plant hydrosols for the first time, corroborating the fascinating phytochemical and antioxidant activity of *Rosmarinus officinalis* hydrosol. This research introduces a new natural biomaterial as a potential source of enzymes, such as lipoxygenase, tyrosinase, and polyphenol oxidase, which could be valuable for environmentally friendly applications in the manufacturing and healthcare industries. A different route for hydrosols’ applications was explored in the study by Chraka et al. [51]. Most organic compounds in hydrosols possess either aromatic or heterocyclic rings with heteroatoms like oxygen. These active sites allow the compounds to form covalent and electrostatic bonds with metal atoms. Leveraging this property, *Thymbra capitata* hydrosol demonstrated cathode-type inhibitor characteristics, making it a promising eco-friendly candidate for more effective and cost-efficient mixed anti-corrosive agents for copper alloy.

Several studies have also examined steam distillation systems. In the research by Ieri et al. [52], the volatile composition of EOs and hydrosols from four *Eucalyptus* species was analysed, revealing chemical differences between the hydrosols and related EOs. GC–MS analysis showed that EOs and hydrosols were primarily composed of oxygenated monoterpenes, with 1,8-cineole being the major component in both. However, *Eucalyptus* EOs exhibited a higher content in monoterpene hydrocarbons than the hydrosols. According to Aćimović et al. [53], in a comparative study of the composition of *Artemisia annua* EO and hydrosol, it was found that the major compounds in the hydrosol (camphor (25.1%), 1,8-cineole (20.5%), and artemisia ketone (10.7%)) were also present in the EO, albeit at lower percentages. Oxygenated monoterpenes were identified as the predominant class of compounds in the hydrosol (89.5%), whereas the essential oil primarily contained oxygenated monoterpenes (53.8%) along with sesquiterpene hydrocarbons (30.9%). In another study [54], a comparison of *Salvia sclarea* EO and hydrosol revealed similarities in their compositions, with linalyl acetate and linalool being the major compounds. This study emphasised the value of this byproduct, particularly concerning sustainability and waste management principles. The study of Aćimović et al. [55] delved into the influence of climatic conditions on the quality of lavandin EOs and hydrosols, revealing that oxygenated monoterpenes were the most prevalent compounds in both products. The authors concluded that the temperature was favourably related to the presence of all compounds. Nevertheless, only the main compounds (linalool, 1,8-cineole and borneol) are positively correlated with precipitation, with the others inversely correlated. Krajewska et al. [56] investigated the correlation between the quantity of hydrosol and raw material (fresh herb) during the distillation to produce high-quality hydrosol by collecting various fractions throughout the process and assessing the chemical composition of the volatile compounds. In addition to several studies devoted to the characterisation of the hydrosols’ volatile profile by GC–MS, Manousi et al. [57] investigated their elemental composition through inductively coupled plasma atomic emission spectrometry (ICP-AES) to evaluate the presence of toxic and nutrient elements such as Ag, Al, B, Ba, Ca, Cd, Co, Cr, Cu, Fe, Mg, Mn, Ni, Pb and Zn. This, avoiding contamination and ensuring consumer safety, is a relevant topic when considering hydrosol safety.

Regardless of the extraction method, hydrosols generally contain more oxygenated monoterpenes than their corresponding EOs. Based on the available data, it can be concluded that the chemical compositions of hydrosols and their respective EOs typically differ quantitatively and often qualitatively. The degree of similarity between the EO and hydrosol composition primarily depends on the EO’s ratio of hydrocarbons to oxygenated compounds. When oxygenated compounds are the predominant component of the EO, the similarity with the hydrosol is very high. Conversely, if hydrocarbons are the main constituents of the EO, the hydrosol composition tends to differ significantly [6,14]. Research comparing HD and SD extraction methods has shown that SD systems produce hydrosols with a more diverse chemical composition, including a higher number of compounds than HD systems. This supports the finding that HD can lead to the degradation of certain compounds through hydrolysis, causing their absence in the final product [7,37,58]. Reviewing the aforementioned studies, it is evident that a higher number used the HD, likely because this technique is more practical to implement at a laboratory scale due to its smaller size requirements. However, studies addressing innovative extraction methods to enhance process efficiency while minimising costs are ongoing, with some reported advantages. Nazlić et al. [59,60], who compared hydrosols from *Veronica* species obtained via two different extraction methods (HD and microwave-assisted water extraction), found that both techniques led to the isolation of the same major compounds. Gonzalez-Rivera et al. [61], in a study comparing simultaneous ultrasound and microwave irradiation-assisted extraction (US-IMWAE) and microwave-assisted extraction (MWAE) with the traditional HD system, concluded that US-IMWAE significantly raised the hydrosols’ total phenolic compound content (172% higher than for HD), thus potentiating higher biological activity. According to Ürgeová et al. [62], hydrosols from *Salvia* species obtained by MWAE showed promising antimicrobial capacity. These findings indicate that exploring hydrosol extraction through these emerging green technologies could be valuable for developing more effective products.

The collected data highlight the critical need for a comprehensive analysis of hydrosols’ chemical compositions and their bioactivity in order to guarantee their safe application in creating new eco-friendly products. It is also important to establish their safety by determining the recommended, non-toxic daily intake for consumers. Another crucial point of volatile compound characterisation concerning the application areas is the need for concentration to enhance hydrosols’ quality and bioactive properties. In this context, Xu et al. [63] explored the use of a polydimethylsiloxane ceramic composite membrane to enrich the volatile aromatic composition of lavender hydrosol. It was possible to achieve hydrosols’ composition concentrations, boosting their antioxidant and biological activity and adding value to these byproducts by adjusting key parameters such as temperature, time, and flow rate during the separation process. These strategies are crucial for the food industry to produce natural aromas, enabling the concentration and recovery of volatile aromatic compounds from, e.g., fruit hydrosols. In a two-article series study [64,65], the pervaporation technique allowed for the recovery and concentration of fruit aromas, with sensory analysis showing an improvement in the aromatic notes and shelf life. The economic analysis suggested that pervaporation is a cost-effective and sustainable method for aroma recovery. Elguea-Culebras et al. [19] presented a compilation of studies assessing the potential of EOs’ distillation residues (solid residue, wastewater, and hydrosols) for recovering phenolic compounds. This study highlighted several promising techniques for processing solid residues, including Soxhlet extraction, water bath extraction, pressurised liquid extraction, microwave-assisted extraction, supercritical CO_2_ extraction, ultrasound-assisted extraction, shaking/stirring extraction, maceration extraction, and the use of the Ultra-Turrax apparatus. It also identified effective methods for treating wastewater, such as evaporation, liquid–liquid extraction, solid–liquid extraction, and pressurised liquid extraction. These findings suggest a sustainable approach for the industrial sector. Additionally, the review revealed that the most abundant compound families in the EOs’ distillation residues were phenolic monoterpenes, diterpenes, hydroxybenzoic acids, phenylpropenes, coumarins, flavanones, flavones, and flavanols. Several studies emphasise the advantages of recovering high molecular weight water-soluble compounds, particularly phenolic compounds, from solid residues and wastewater, as these compounds are closely linked to bioactivity manifestation [61,66,67,68,69,70,71].

Different characterisation methods are being proposed as the specifications for hydrosol standardisation still require improvement. Table 1 summarises the bibliographic survey results on the chemical characterisation methods applied to hydrosols produced by different processes and conditions. The data were catalogued by vegetal matrix, the used extraction procedure and parameters, namely plant/water ratio (*w*/*v*), distillation time, apparatus, and solvent in the LLE step. The identified compounds are within the following classes: terpene oxides (e.g., 1,8-cineole), phenolic monoterpenoids (e.g., thymol, carvacrol, eugenol), monoterpenoid alcohols (e.g., terpinen-4-ol, linalool, geraniol), monoterpenes (e.g., limonene, a-pinene, ocimene), monoterpene aldehydes (e.g., citronellal, neral, geranial, cinnamaldehyde), cyclic monoterpene ketone (e.g., carvone, camphor) and phenylpropene [72].

### 3.3. Biological Characterisation

The diverse bioactivities of hydrosols are mainly associated with the chemical composition of their volatile compounds [9,80]. These compounds are byproducts of the plant’s defence metabolism. They can be categorised into three major chemical groups: terpenes, phenolic compounds, and alkaloids, with their activity influenced by the presence of specific functional groups within their molecular structure. According to the reported studies, phenolic compounds, including terpenes, phenols, esters, aromatic compounds, aldehydes, or terpenoids, are the main antibacterial agents responsible for inhibiting microorganism growth. In addition, other oxygen monoterpenoid compounds, like aldehydes, ketones, and esters, are associated with the antioxidant activity of hydrosols [81,82]. Thus, in addition to chemical profile determination, bioactivity assessment is also an important aspect to consider when evaluating the potential of hydrosols. Furthermore, gaining insight into their mechanisms of action and identifying the compounds responsible for the various bioactive properties is highly important.

Hydrosols are traditionally utilised for various purposes due to their wide range of valuable properties, including pleasant scents, relaxing effects, and antimicrobial properties. Their applications span multiple industries, such as food and beverages (as flavouring or preservative additives), cosmetics (as a replacement for the water phase in cosmetic products), aromatherapy (in perfumery, skincare, and massage products for relaxation), biological agriculture (as biocides), and medicine (as potential anti-tumour agents and natural antibiotics), making them popular worldwide. Currently, hydrosols are being investigated for their eco-friendliness, health benefits, and safety characteristics. A comparison of their antimicrobial and antioxidant properties and their chemical composition is performed against their corresponding EOs [9,16,83].

#### 3.3.1. Antioxidant Activity

A high concentration of free radicals in foods can cause damage and thus degradation phenomena like lipidic peroxidation or enzymatic browning. The free radicals’ production depends on the natural environment, external factors, or the substance’s metabolism, leading to oxidative stress. In this context, plant compounds have raised attention due to their promising antioxidant potential and ability to delay the oxidative process. Antioxidant evaluation can be assessed based on radical termination through two mechanisms: hydrogen atom transfer (HAT), which involves neutralising free radicals by donating a hydrogen atom, and single electron transfer (SET), which measures the ability of an antioxidant to transfer one or more electrons to reduce oxidised radicals. This capacity is quantified through various mechanisms, as follows: chain initiation, free radical scavenging, reducing power, peroxide termination, prevention of ongoing hydrogen extraction, and inhibition of singlet oxygen formation. The cell antioxidant activity assay was developed to assess the response at the cellular level [80,84,85]. In summary, these mechanisms involve the interaction between antioxidant agents and reactive oxygen species (free radicals) to prevent or reduce oxidative stress. 

Several studies on the antioxidant activity of hydrosols are available in the literature. Merad Boussalah [86] investigated *Cynoglossum cheirifolium* hydrosols and reported notable levels of antioxidant activity, as demonstrated by DPPH free radical scavenging and linoleic acid oxidation tests. This bioactive potential was attributed to the high content of oxygenated compounds, with 2-pentyl-furan and carvone being the most prominent compounds. Değirmenci et al. [11] investigated the antioxidant activity of *C. aurantium* hydrosol using DPPH free radical scavenging and hydrogen peroxide scavenging assays. The results reveal excellent antioxidant potential, attributed to the high levels of monoterpene and sesquiterpene compounds (such as linalool, α-terpineol, hotrienol, nerol, nerolirol, and farnesol). These compounds can interrupt free radical chain reactions and lead to their irreversible oxidation into inert substances.

#### 3.3.2. Antimicrobial Activity

Microbial contamination of food by bacteria, fungi, viruses, parasites, and other microorganisms is a significant global concern. Plant-derived compounds with antibacterial and/or antifungal properties can be employed as antimicrobial agents to prevent food spoilage and foodborne illnesses. Their effectiveness often depends on the chemical structure and the presence or site of specific functional groups. The mechanism of antimicrobial action may differ among different bioactive compounds [80,87,88]. These mechanisms depend on the bioactive compounds and microorganisms’ characteristics, as well as on environmental stresses. From a general perspective, these mechanisms consist of cell wall/membrane disruption, which affects essential metabolic functions like energy production, respiration and genetic material production. Kachur et al. [89] investigated different mechanisms for the antimicrobial activity of thymol and carvacrol (both compounds found in some hydrosols), corroborating their ability to act in the disruption of the bacterial membrane and inhibit efflux pumps, bacterial motility, and membrane ATPases. According to Jeyakumar and Lawrence [90], eugenol presented bactericidal action against *Escherichia coli*, showing a mechanism whereby the compound acts on the cell membrane, modifying its permeability and leading to the leakage of intracellular contents, causing cell damage.

Several researchers have studied the biological activities of hydrosols obtained from different plants, including antimicrobial, antifungal, anti-inflammatory, and antioxidant activities. According to Belabbes et al. [91], the EO and the hydrosol extracted from *Calendula arvensis* L. demonstrated excellent antioxidant and antifungal activity. These properties were related to their chemical profile, particularly the high percentage of oxygenated sesquiterpenes. The results of both in vivo and in vitro antifungal tests showed that hydrosols can reduce growth and control infections, showing potential to be exploited in pear fruit treatment. Bellahsene et al. [92] concluded that the EO and hydrosols obtained from *Nepeta nepetella* subsp. amethystine showed potent antimicrobial activity against bacteria and moulds. Hydrosol antimicrobial activity was related to nepetalactone, its major monoterpenoid component. Di Vito et al. [15] conducted a comparative study of the EO and respective hydrosol antimicrobial activity, reporting that hydrosols can be more effective in inhibiting microbial growth due to the higher antimicrobial activity at lower concentrations. This result indicates that the volatiles are more effective when present in the hydrosol. This may be due to their higher hydrophilicity and aqueous environment, which increase bioavailability for interaction with microorganisms. Another study [93] evaluated the antioxidant and antimicrobial potential of *Thymus satureioidis*, *Anviella radiata*, *Warionia saharae*, *Rosa damascena*, and *Artemisia herba alba* hydrosols. The *T. satureioidis* hydrosol had the highest phenolic concentration and antioxidant activity, inhibiting the growth of *Candida albicans*, *Staphylococcus aureus* and *E. coli.* Bioactivities could be attributed to the presence of borneol, carvacrol and thymol in its composition. In a more recent study, Di Vito et al. [94] developed a green formulation (composed of 0.03% of *Cinnamomum zeylanicum* EO in 99.97% of *Citrus aurantium* hydrosol) as an alternative to biocides normally used in the restoration of artworks, showing that the formulation presented an effective antifungal potential (concentration of 28 μL/cm^2^, 5 h treatment). Another study [95] has reported that *Thymbra spicata* hydrosol was less effective than its corresponding EO as an antimicrobial agent. When stimulated with UV light, the hydrosol efficacy increases, becoming highly potent against *S. aureus*, activity that is attributed to thymol and carvacrol. Exploring synergistic effects can thus enhance the use of hydrosols across various industries, such as UV hygiene in cosmetics, packaging, and pharmaceuticals. Santarsiero et al. [96] found that *Pistacia lentiscus* hydrosol demonstrated anti-inflammatory activity by inhibiting secretions in lipopolysaccharide-activated primary human monocytes. This effect was attributed to the presence of certain metabolite classes (like anti-inflammatory sulphur-bearing peptides), indicating its potential as a therapeutic agent.

## 4. Applications

This section concerns hydrosol applications and is organised by the following key fields: food applications (including their use as natural additives, sanitisers, food enrichments, and browning prevention), agricultural applications, pharmaceutical applications, medicinal applications, and cosmetic applications.

### 4.1. Food Applications

Apart from consumption as functional beverages [9,10,11], as discussed in the previous sections, this byproduct can serve as a natural food additive with the potential for industrial-scale use.

#### 4.1.1. Hydrosols as Natural Additives in the Food Industry

Natural preservatives can be used for food preservation, being differentiated by their action mechanism. Antioxidant agents are used as preservatives because they limit or delay food deterioration, preventing the auto-oxidation of lipids, pigments, flavours, and vitamins. Antimicrobial agents control and prevent the deterioration imparted by microorganisms [97]. A survey of studies on hydrosols as natural additives is summarised in Table 2. The bioactivity of hydrosols obtained by HD was reported in several studies. According to Khan et al. [98], *Origanum vulgare* hydrosol revealed potent antibacterial activity against the strains *S. aureus*, *M. luteus*, *E. coli* and *P. aeruginosa*, attributed to its main compounds (carvacrol, thymol and terpinen-4-ol), with carvacrol, the dominant compound, exhibiting the highest activity. Based on these findings, the authors claim that hydrosols could be a promising alternative to traditional food preservatives. Culmone et al. [99] have developed an edible coating containing *Origanum vulgare* hydrosol for treating papaya fungal infections. This treatment showed antimicrobial activity without changing the fruit’s organoleptic characteristics, suggesting hydrosols as valuable natural and safe agents for extending a fruit’s shelf life. Another study [100] found that the high levels of thymol and carvacrol in *Thymus* species’ hydrosols gave them a higher biological potential than their corresponding EOs. These hydrosols also exhibited significant antibacterial activity against Gram-positive and Gram-negative bacteria, making them an excellent alternative to synthetic food preservatives. Yavuzer et al. [101] investigated hydrosols from orange peel, pomegranate peel, shaddock peel, mandarin peel, and thyme as alternative antimicrobial agents against common foodborne and fish spoilage bacteria. They found that the antimicrobial activity varied depending on the tested bacterial strains, with thyme and pomegranate peel hydrosols showing the strongest performance. Another study by the same group [102] demonstrated that atomised thyme and lavender hydrosol droplets applied to meat effectively preserved food quality over time by reducing bacterial growth and oxidation levels without compromising sensory quality. In addition, Zaccardelli et al. [17], who investigated hydrosols from basil, rosemary and sage, concluded that their practicality and ease of use are attributed to their favourable physicochemical properties and potential antioxidant activity.

Furthermore, studies concerning SD systems were also investigated. Gharb et al. [103] investigated the antioxidant activity of *Eucalyptus camaldulensis* hydrosol, uncovering its potential as a natural antioxidant for use in the cosmetic industry and food preservation. Aćimović et al. [104] reported the chemical composition and bioactivities (antioxidant and antimicrobial) of *Dracocephalum moldavica* hydrosol, demonstrating that geranial, commonly utilised as a flavouring agent in food and beverage products, is the predominant compound. Erceg et al. [105] developed an edible coating using *Cymbopogon citratus* and *Helichrysum italicum* hydrosols to extend the shelf life of sliced cheese. This coating, which combines hydrosols with biopolymers (pullulan–chitosan and pullulan–gelatine), demonstrated antimicrobial effectiveness against *S. aureus*. The bilayer coatings restricted bacterial growth in pre- and post-application contamination scenarios, emphasising the antimicrobial properties of the hydrosols. The pullulan–chitosan formulation with hydrosols exhibited a biocidal effect for contaminations before coating, while the pullulan–gelatine formulation provided a long-term bacteriostatic effect. Both coatings effectively inhibited bacterial growth regardless of contamination timing, highlighting their potential for enhancing food safety. In another study [106], the antimicrobial activity against *Salmonella enterica* of eight hydrosols obtained by HD at the laboratory scale (basil, calendula, oregano, corn silk, laurel, rosemary, spearmint, and thyme) was compared with an industrial SD hydrosol (oregano). The study found that these aqueous byproducts could serve as eco-friendly natural antimicrobial agents without compromising food sensory properties. The SD industrial oregano hydrosol exhibited the highest antibacterial potential, effectively eradicating *Salmonella* strains (~10^6^ CFU/mL) immediately after inoculation. During incubation at 4 °C, HD extracts reduced or eliminated *Salmonella* levels. At 37 °C, oregano, centrifuged oregano, thyme, calendula, and basil hydrosols were bactericidal, while spearmint, rosemary, and corn silk hydrosols were bacteriostatic. The superior antibacterial activity of the industrial oregano hydrosol is likely due to its high carvacrol content. This study provides valuable insights by comparing laboratory and industrial scales hydrosols, emphasising their potential bioactive properties and potential industrial applications.

**Table 2:** Survey of different hydrosols as natural additives for food application.

Plant Scientific Name	Extraction and Characterisation Methodologies	Main Results	Reference
*Psoralea bituminosa*	Extraction procedure: HD; Chemical composition: GC–MS; Biological characterisation: antioxidant activity (DPPH, FRAP and β-carotene bleaching assays).	Major compounds: caryophyllene oxide (35.5%) and E-phytol (25.6%); Antioxidant activity: for DPPH (IC_50_ = 2.60 mg/L), FRAP (0.50–1.75 mg/L), and β-carotene inhibition (IC_50_ = 0.32 mg/L).	[26]
*Areca catechu* and *Cocos nucifera*	Extraction procedure: HD; Chemical composition: GC–MS; Biological characterisation: antioxidant activity (total phenolic content, reducing power, DPPH and ABTS assays), antibacterial activity (agar disc diffusion and serial dilution methods).	Major compounds:*A. catechu* hydrosols (benzyl alcohol (14.39%), 1-heptanol (13.84%), ethyl-2-(5-methyl-5-vinyltetrahydrofuran-2-yl)propan-2-yl (13.27%)), *C. nucifera* hydrosol (ethyl-2-(5-methyl-5-vinyltetrahydrofuran-2-yl)propan-2-yl (11.62%)); Antioxidant activity: for *A. catechu* hydrosols (0.093 for reducing power, 81.83% inhibition for DPPH assay and 58.60% inhibition for ABTS assay), *C. nucifera* hydrosols (0.122 for reducing power, 71.32% inhibition for DPPH assay and 63.57% inhibition for ABTS assay); Antibacterial activity: inhibition zone diameter of *A. catechu* hydrosols (11.2 mm on *E. coli*, 12.1 mm on *C. albicans*, 10.2 mm on *E. coli* O157:H7, 12.7 mm on *S. aureus*), of *C. nucifera* hydrosols (13.8 mm on *E. coli*, 14.2 mm on *C. albicans*, 6.8 mm on *E. coli O157:H7*, 12.3 mm *on S. aureus).*	[107]
*Lippia alba*, *Rosmarinus officinalis* and *Thymus vulgaris*	Extraction procedure: SD; Chemical composition: GC–FID and GC–MS; Biological characterisation: antioxidant activity (ABTS assay), antimicrobial activity (microplate and agar dilution methods).	Major compounds: for *L. alba* hydrosol (carvone (92.7%)); *R. officinalis* hydrosol (1,8-cineole (38.2%), and camphor (51.9%)); *T. vulgaris* hydrosol (thymol (98.1%)); Antioxidant activity: for T. vulgaris* hydrosol (IC_50_ = 3038.10 μL/L); Antimicrobial activity: T. vulgaris* hydrosol showed microbicide effect on *P. aeruginosa*, *S. aureus*, *C. albicans*, and *A. niger;*	[108]
*Origanum vulgare* L.	Extraction procedure: HD; Chemical composition: GC–FID and GC–MS; Biological characterisation: antimicrobial activity (optical density of cells at 600 nm).	Major compounds: carvacrol (92.5%); Antimicrobial activity: IC_50_ values (μg/mL) of 107 on *S. aureus*, 174 on *M. luteus*, 127 on *E. coli*, and 286 on *P. aeruginosa*	[98]
*Cinnamomum Verum*	Extraction procedure: SD (commercial sample); Chemical composition: GC–MS; Biological characterisation: antimicrobial activity (agar disc diffusion method).	Major compounds: vulgarol, emersol, cinnamic acid methyl, methyl palmitate and oleic acid (% not mentioned); Antimicrobial activity: inhibition zone diameter > 30 mm on *S. aureus* and *S. saprophyticus.*	[109]
*Daphne gnidium* L.	Extraction procedure: HD; Chemical composition: GC–FID and GC–MS; Biological characterisation: antioxidant activity (FRAP and DPPH assays), antimicrobial activity (agar disc diffusion method).	Major compounds: carvone (10.9%) and carvacrol (10.9%); Antioxidant activity: for FRAP (0.42 mg AAE/mL) and DPPH (IC_50_ = 0.62 mg/mL); Antimicrobial activity: MIC values (μL/mL) of 31.35 on *S. aureus*, 62.5 on *E. feacalis*, 15.62 on *B. cereus*, 3.90 on *B. subtilis*, 7.81 on *E. coli*, 7.81 on *P. aeruginosa*, and 15.62 on *K. pneumoniae.*	[110]
*Centaurea cyanus*, *Citrus aurantium*, *Jasminum grandiflorum*, *Lavandua angustifólia*, *Matricaria chamomilla*, *Rosa centifólia* and *Rosa damascena*	Extraction procedure: not mentioned (commercial sample); Chemical composition: not investigated; Biological characterisation: antioxidant activity (FRAP assay, cytotoxicity, and DNA damage level).	Antioxidant activity: hydrosols revealed the lowest antioxidant power; Impact on cell viability: hydrosols were excluded due to its weak activity and lack of standardisation during preparation.	[111]
*Rosa alba* and *Rosa damascena*	Extraction procedure: HD; Chemical composition: GC–MS; Biological characterisation: antioxidant activity (total phenolic content, TBARS, DPPH and superoxide anion radicals generating system methods).	Major compounds:*R. damascena* hydrosols (β-citronellol (28.70%), *trans*-geraniol (16.44) and *cis*-geraniol (10.81%)), *R. alba* hydrosols (*trans*-geraniol (36.44%) and β-citronellol (28.69%)); Antioxidant activity: *R. damascena* hydrosols (20% TBARS inhibition at 15% concentration, approximately 40% radical scavenging effect, -OH and O^−2^, at 1.25% concentration and 9.37% concentration, respectively), *R. alba* hydrosols (22% TBARS inhibition at 15% concentration, approximately 30–38% radical scavenging effect, OH and O^−2^, at 6.25% concentration and 9.37% concentration, respectively).	[112]
*Eucalyptus camaldulensis*	Extraction procedure: SD; Chemical composition: not investigated; Biological characterisation: antioxidant activity (DPPH and FRAP assays).	Antioxidant activity: adult leaves revealed higher activity (400, 600 and 800 μg/mL) for both methods.	[103]
*Cynoglossum cheiriforium* L.	Extraction procedure: HD; Chemical composition: GC–MS; Biological characterisation: antioxidant activity (DPPH and β-carotene/linoleic acid assays), antifungal activity (agar disc diffusion method).	Major compounds: 2-pentyl-furan (46.3%) and carvone (23.5%); Antioxidant activity: for DPPH (IC_50_ = 18.2 μL/mL) and for the β-carotene/linoleic acid method (1.2 μL/mL) Antifungal activity: at concentration of 30 μL/mL an inhibition percentage of 100% was observed against *A. alternata*, 93.4% on *P. expansum*, and 89.2% on *A. niger*.	[86]
*Citrus sinensis*, *Punica granatum*, *Citrus maxima*, *Thymus vulgaris* and *Citrus reticulate*	Extraction procedure: HD; Chemical composition: not investigated; Biological characterisation: antimicrobial activity (microdilution and agar disc diffusion methods).	Antimicrobial activity: MIC values of hydrosols against foodborne bacteria (*S. aureus*, *Salmonella* Parathyphi, and *K. pneumoniae*) was generally 50 mg/mL; MIC values of hydrosols against fish spoilage bacteria (*V. vulnificus*, *P. luteola*, and *P. damselae*) was also 50 mg/mL, with exception of thyme hydrosol that showed MIC value of 25 mg/mL on *V. vulnificus*.	[101]
*Phyllostachys heterocycle*	Extraction procedure: HD; Chemical composition: GC–MS; Biological characterisation: antimicrobial activity (agar well diffusion assay, antimicrobial stability assay, cell membrane integrity).	Major compounds: ionone (16.76%) and β-damascenone (10.38%); Antimicrobial activity: MIC values of ¼ concentration (*v/v*) on *S. aureus* and *B. subtilis*, and ½ concentration (*v/v*) on *E. coli* and *S. cerevisiae*; Antimicrobial stability tests: hydrosols exhibited good stability under heat treatment, change in pH, and exposure to UV radiation; Cell membrane integrity: hydrosols destroyed the cell wall and the cell membrane permeability.	[113]
*Eucalyptus camaldulensis*	Extraction procedure: SD; Chemical composition: not investigated; Biological characterisation: antimicrobial activity (microtiter plate method).	Antibacterial activity: concentration of 25% of hydrosol inhibited *S. pyogenes*, and 100% of hydrosol inhibited *E. coli*, *K. pneumoniae*, *P. aeruginosa*, *P. vulgaris*, *S. aureus*, *S. epidermidis*, and *S. mutans.*	[114]
*Litsea cubeba*	Extraction procedure: SD; Chemical composition: GC–MS; Biological characterisation: antimicrobial activity (broth dilution assay and time–kill kinetics assay).	Major compounds: geranial (32.92%) and neral (27.12%); Antimicrobial activity: MIC and MBC values (*v/v*) against *H. pylori* (10 and 30% hydrosol, respectively); MIC and MBC values (*v/v*) against *C. albicans* (10 and 40% hydrosol, respectively); Time–kill kinetics assay: *H. pylori* and *C. albicans* cells were killed after 14–18 h of treatment with 30 and 40% hydrosol, respectively.	[83]
*Canarium ovatum*	Extraction procedure: HD and SD combination; Chemical composition: GC–FID and GC–MS; Biological characterisation: antibacterial activity (microdilution method).	Major compounds: α-phellandrene oxidation products including *cis*-α-phellandrene epoxide and a series of *para*-menth-5-ene-1,2-diol isomers (% not mentioned); Antibacterial activity: MIC_90_ values (mg/mL) of 0.26–0.45 on *E. coli* and 0.20–0.44 on *S. aureus.*	[115]
*Citrus aurantium* L.	Extraction procedure: SD; Chemical composition: GC–MS; Biological characterisation: antioxidant activity (total phenolic and flavonoid content, DPPH and H_2_O_2_ scavenging methods), antimicrobial activity (agar disc diffusion and broth microdilution methods).	Major compounds: linalool (16.58%); Antioxidant activity: IC_50_ values of 384.33 μg/mL for DPPH assay, 354.99 μg/mL for H_2_O_2_ percent scavenging; Antimicrobial activity: hydrosol did not show antimicrobial activity within the studied range of concentrations.	[11]
*Thymus* sp., *Myrtus communis* L., *Eucalyptus globulus* L. and *Rosmarinus officinalis* L.	Extraction procedure: not mentioned (commercial samples); Chemical composition: not investigated; Biological characterisation: antioxidant activity (DPPH and ABTS assays, total phenolic and flavonoid content), antimicrobial activity (agar disc diffusion method).	Antioxidant activity: DPPH and ABTS radical scavenging activities exhibited a dose-dependent manner and increased with increasing concentrations; Antimicrobial activity: thyme* hydrosol presented an inhibition zone diameter ranging from 8–13 mm on *S. aureus*, *E. faecalis*, *G. rubripertincta*, *E. aerogenes*, *Salmonella enterica*, *P. vulgaris*, and *K. pneumoniae* and inhibited fungi ranging from 9–11 mm on *C. albicans*, *C. tropicalis* and *C. parapsilosis.*	[116]
*Laurus nobilis*, *Salvia officinalis* and *Salvia slarea*	Extraction procedure: SD; Chemical composition: GC–MS; Biological characterisation: antioxidant activity (DPPH and ABTS assays), antimicrobial activity (microdilution and agar disc diffusion methods).	Major compounds:*L. nobilis* hydrosol (1,8-cineole (65.1%) and α-thujone (11.1%)); *S. officinalis* hydrosol (1,8-cineole (61.4%) and camphor (22.5%)); *S. sclarea* hydrosol (linalool (89.5%) and α-terpineol (10.5%)); Antioxidant activity: for DPPH and ABTS assay, IC_50_ values (μg/mL) of 218.10 and 391.38, from *L. nobilis* hydrosol, respectively; of 135.58 and 551.38, from *S. officinalis* hydrosol, respectively; and of 200.43 and 479.27, from *S. sclarea*, respectively; Antimicrobial activity: hydrosols showed no activity at the studied concentrations against the bacterial strains.	[117]
*Rosmarinus officinalis* and *Lavandula angustifolia*	Extraction procedure: SD (commercial sample); Chemical composition: GC–FID and GC–MS; Biological characterisation: antioxidant activity (DPPH and ABTS assays), cytotoxic activity (MTT test), antibacterial activity (agar disc diffusion method and vapor phase test).	Major compounds:*R. officinalis* hydrosol (1,8-cineole (56.2%), camphor (20.3%) and borneol (10.6%)); *L. angustifolia hydrosol* (linalool (42.9%), camphor (18.4%), α-terpineol (12.6%), 1,8-cineole (11.8%)); Antioxidant activity: for DPPH and ABTS, IC_50_ values (μg/mL) of 136.30 and 349.42 for *R. officinalis* hydrosol, respectively and of 240.02 and 181.24 for *L. angustifolia* hydrosol, respectively; Cytotoxic activity: EC_50_ value at 24 h of 26.82% for *R. officinalis* and 30.18% for *L. angustifolia* hydrosols on human neuroblastoma cells; Antibacterial activity: hydrosols showed no activity at the studied concentrations against the bacterial strains.	[118]
*Piper longum* Linn.	Extraction procedure: solvent-free microwave extraction system; Chemical composition: GC–MS; Biological characterisation: antioxidant activity (DPPH, ABTS and reduction power assays).	Major compounds: eugenol (25.44% and 22.82%, SFME and HD procedure), linalool (14.10% and 22.37%, SFME and HD procedure); Antioxidant activity: for DPPH (IC_50_ values (mg/mL) of 6.31 and 49.47 for SFME and HD hydrosols, respectively), for ABTS (IC_50_ values (mg/mL) of 5.53 and 12.43 for SFME and HD hydrosols, respectively) and for reducing capabilities, both hydrosols were significantly higher when compared with the control.	[20]
*Thymus vulgaris*, *Thymus pannonicus*, *Lavandula angustifolia*, *Lavandula x intermedia*, *Origanum vulgare* and *Origanum vulgare* var. *aureum*	Extraction procedure: SD; Chemical composition: GC–MS; Biological characterisation: antioxidant activity (DPPH and ABTS assays, total phenolic content).	Major compounds:*T. vulgaris* hydrosol (thymol (62.96%) and carvacrol (21.48%)); *T. pannonicus* hydrosol (geranial (37.91%) and β-citral (27.7%)); *L. angustifolia* hydrosol (linalool (22.47%), eucalyptol (20.8%), and camphor (16.94%)); *Lavandula x intermedia* hydrosol (linalool (29.83%), eucalyptol (24.38%), endo-borneol (13.65%), and terpinen-4-ol (10.45%)); *O. vulgare* hydrosol (1-octen-3-ol (13.31%) and linalool (11.59%)); *O. vulgare* var. *aureum* hydrosol (linalool (54.09%), and thymol (21.96%)); Antioxidant activity: the hydrosol inhibition results ranged from 4.89 to 16.97% for DPPH assay, and from 10.11 to 98.16% for ABTS assay. *T. vulgaris* hydrosol presented the highest inhibition for both methods.	[119]
*Dracocephalum moldavica*	Extraction procedure: SD; Chemical composition: GC–FID; Biological characterisation: antioxidant activity (DPPH, ABTS, superoxide anion, β-carotene bleaching and reducing power assays), antimicrobial activity (agar disc diffusion method).	Major compounds: geranial (23.4%), neral (22.4%), and geraniol (21.3%); Antioxidant activity: for DPPH (8.82 μmol/100 mL), for ABTS (25.44 μmol/100 mL), for superoxide oxide (19.58 μmol/100 mL), for β-carotene bleaching (41.63 μmol/100 mL), and for reducing power (9.50 μmol/100 mL); Antimicrobial activity: inhibition zone diameters of 11.0 mm on *B. cereus*, 28.3 mm on *S. aureus*, 27.0 mm on *L. monocytogenes*, 34.3 mm on *E. coli*, and 10.3 mm on *Salmonella*.	[104]
*Origanum vulgare* var. *hirtum* and *Coridothymis capitatus*	Extraction procedure: not mentioned (commercial samples); Chemical composition: methodology not mentioned; Biological characterisation: antimicrobial activity (broth microdilution technique and time–kill kinetics assay).	Major compounds: *O. vulgare* hydrosol (thymol (100%)); *C. capitatus* hydrosol (carvacrol (100%)); Antimicrobial activity: both hydrosols’ MIC values ranged from 125 to 500 μL/mL on different *L. monocytogenes* strains; the time–kill kinetics assay showed that hydrosols significantly decreased the viable cell numbers of *L. monocytogenes* in a time-dependent and strain-dependent manner (in 60 min exposure, both hydrosols reduced the cellular load of each tested strain by about 1.2–1.7 log CFU/mL).	[120]
*Cupressus leylandii*, *Eucalyptus globulus*, *Aloysia citrodora* and *Melissa officinalis*	Extraction procedure: HD; Chemical composition: GC–MS; Biological characterisation: antimicrobial activity (viable cell counting method).	Major compounds: *C. leylandii* hydrosol (terpinene-4-ol (36.20%) and oplopanonyl acetate (12.76%)); *E. globulus* hydrosol (1,8-cineole (90.12%)); *A. citrodora* hydrosol (neral (39.01%) and geranial (38.91%)); *M. officinalis* hydrosol (neral (42.03%) and geranial (50.08%)); Antimicrobial activity: the results show that hydrosols antimicrobial potential increased with concentration. The most promising ones were *A. citrodora* > *M. officinalis* > *E. globulus* > *C. leylandii.* At 20% (*v/v*) concentration *A. citrodora* hydrosol inhibited 90% of *E. coli* and 80% of *C. albicans* growth. Additionally, M. officinalis* hydrosol significantly reduced growth by 70.0% for *S. aureus* and 79.6% for *C. albicans*, while E. globulus* hydrosol reduced *C. albicans* growth by 71.3%.	[5]

HD = hydro distillation system; GC–MS = gas chromatography–mass spectrometry; DPPH = free radical scavenging assay; FRAP = ferric-reducing antioxidant power assay; IC_50_ = concentration needed to inhibit 50% of the oxidant species (for antioxidant activity); SD = steam distillation system; GC–FID = gas chromatography with diode array detection; ABTS = free radical scavenging assay; IC_50_ = concentration needed to inhibit 50% of the pathogen (for antimicrobial activity); HPLC = high-performance liquid chromatography; AAE = ascorbic acid equivalent; MIC = minimal inhibitory concentration; MBC = minimal bactericidal concentration; DNA = deoxyribonucleic acid; UV = ultraviolet radiation; MIC_90_ = concentration inhibiting 90% of bacterial proliferation; MTT = thiazolyl blue tetrazolium bromide test; EC_50_= half maximal effective concentration; SFME= solvent-free microwave-assisted extraction; HD = hydro distillation; CFU = colony forming unit; * indicates the plant hydrosols with the most promising results.

#### 4.1.2. Hydrosols as Natural Sanitisers

**Figure 4:**
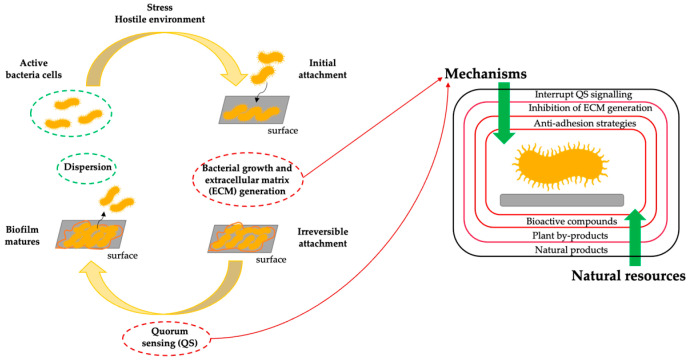
Biofilm life cycle representation and novel antimicrobial strategies for the prevention of biofilm formation (adapted from [123,124]).

This section will address using hydrosols as natural sanitisers for food safety and hygiene applications. Food safety is a major concern in the food industry, particularly due to bacterial biofilm formation, which can lead to cross-contamination and reduced shelf life. Biofilms represent the most resistant form of bacterial growth, providing protection and survival of bacteria in hostile environments and increasing their resistance to antimicrobial agents. The initial step in biofilm formation is the microorganism’s adhesion to various materials and surfaces (see Figure 4). Therefore, modulating bacterial adhesion and implementing efficient sanitisation strategies are crucial for controlling and reducing pathogens in food-processing environments [121,122,123]. After analysing the biofilm life cycle, important factors preventing bacterial attachment and growth can be identified. Bioactive compounds present in natural products can promote inhibition and anti-adhesion effects through different mechanisms. Figure 4 also illustrates the mechanisms by which natural compounds can impede biofilm formation, highlighting their potential as a novel strategy for biofilm control.

The anti-biofilm potential of natural compounds encompasses the inhibition of the polymer matrix formation, suppression of cell adhesion and interruption of the extracellular matrix (ECM) attachment, decreasing the virulence production factors, and thereby blocking the formation of a quorum sensing (QS) network and biofilm development [124]. The initial stages are extremely important, particularly the adhesion stage, which involves cytoskeletal elements such as flagella, fimbriae and the production of lipopolysaccharides. This is followed by forming a QS network within the bacterial group, essential in biofilm formation. Therefore, targeting one or both stages is vital for inhibiting biofilm development [125]. Bioactive compounds can act through different mechanisms, as follows: (i) anti-adhesion strategies, as the adhesion stage is primarily influenced by surface properties such as electrostatic charge and hydrophobicity, bacterial surface attachments, and environmental factors like temperature, exposure time, and nutrient composition; (ii) inhibition of ECM production by disrupting the generation of compounds such as DNA, proteins, carbohydrates, thereby preventing biofilm maturation, coaggregation, and efflux pump activity; (iii) disruption of QS signalling by blocking cell-to-cell communication, either through the inhibition and degradation of signalling molecules or by mimicking these signals to prevent their binding to receptors [124,125,126,127].

Hydrosols can be applied as natural sanitisers on food preparation surfaces or directly on food products to avoid microbial growth, contributing to food safety. Table 3 summarises the gathered information concerning hydrosol applications as natural sanitisers in the food industry. For HD systems, Karampoula et al. [128] have reported that the hydrosol derived from *Thymbra capitata* is a highly effective antimicrobial agent against both planktonic and biofilm cells of *Salmonella*, outperforming a common industrial disinfectant. Moreover, it can be easily rinsed off surfaces and avoids the strong odour associated with essential oils. The primary component of *T. capitata* hydrosol is carvacrol, which has demonstrated significant bacteriostatic and bactericidal activity against foodborne microorganisms. Xylia et al. [129] have assessed the antimicrobial effectiveness of various washing treatments using aqueous solutions of mint (*Mentha piperita*) hydrosol (1:10) against two major foodborne pathogens, *E. coli* and *Listeria monocytogenes*, on shredded carrots. They concluded that mint hydrosol effectively preserved the quality and safety of the carrots, highlighting its potential as an alternative to chlorine for the partial disinfection of minimally processed fresh products. Ozturk et al. [130] demonstrated that hydrosols could serve as natural sanitisers for fresh-cut lettuce, offering effective decontamination. Hydrosols from sideritis, bay leaf, thyme, summer savoury, oregano, rosemary, and salvia showed promising results in reducing pathogen levels on lettuce, specifically in the cases of *E. coli*, *Listeria monocytogenes*, and *Salmonella Typhimurium* contamination. Bezek et al. [131] reported the antimicrobial and anti-adhesion potential of *Helichrysum italicum* hydrosol against four foodborne pathogens (*C. jejuni*, *E. coli*, *P. aeruginosa*, and *S. aureus*), highlighting it as a promising eco-friendly disinfecting alternative for abiotic surfaces.

Hydrosols obtained through SD have also shown potential as sanitisers. Acimovic et al. [132] studied the effects of six hydrosols (peppermint, oregano, fennel, hop, lavender, and lemon catmint) on alfalfa seed germination, antimicrobial activity, and sensory characteristics. The study found that peppermint and fennel hydrosols effectively ensured food safety and improved sensory perceptions. Furthermore, hydrosols already marketed in Italy with food-grade status were investigated. Buccioni et al. [133] assessed the antimicrobial effectiveness of commercial *C. capitatus* hydrosol against *L. monocytogenes* under various relevant food environmental conditions, such as different carbon sources, pH, and sodium chloride presence (1–8%). The study demonstrated that pre-exposure of the cells to the hydrosol induced significant changes in membrane hydrophobicity and cellular spatial distribution, leading to cellular auto-aggregation as a stress response mechanism. These findings suggest that the *C. capitatus* hydrosol shows potential to be used as a sanitiser in food applications or processing environments. Purgatorio et al. [134] examined the use of commercial *T. capitata* hydrosol as a washing solution for rocket salad stored at 4 °C for 48 h. The hydrosol effectively reduced the levels of both inoculated *L. monocytogenes* (circa 1 log CFU/g) and naturally occurring microorganisms after a treatment of 5 min without adversely affecting the salad pH, water activity, colour, and sensory quality. Another study [135] investigated the antiviral efficacy of commercial *Citrus limon* and *Thymus serpyllum* hydrosols in oyster depuration. The authors have reported a notable reduction of 0.2 log in norovirus (NoV) concentration after 24 h of treatment with 1% *C. limon* hydrosol, corroborating the effectiveness of this hydrosol-based post-harvest treatment. This method could be a promising approach to enhance oysters’ sensory qualities and food safety. These studies collectively demonstrate that hydrosols hold potential as sustainable sanitising agents. Additionally, the use of commercial hydrosols highlights their market potential as value-added products due to their bioactive, non-toxic, and eco-friendly attributes.

#### 4.1.3. Hydrosols as Food Enrichments

Most published studies primarily focus on assessing the potential of byproducts rather than evaluating their use in final applications. However, hydrosols offer more than just food sanitising capabilities. They can be directly incorporated into food formulations to create enriched products. By using hydrosols as an ingredient, the bioactive potential of food products can be enhanced. Table 4 outlines several studies exploring hydrosols’ use for food enrichment. Kakoei et al. [136] sought to create a yoghurt drink infused with *Eryngium caucasicum* hydrosol, extracted by microwave-assisted, ultrasound-assisted, and ultrasound microwave-assisted hydro distillation. The resulting yoghurt drink demonstrated remarkable antioxidant properties attributed to its bioactive components. Another study [137] examined the impact of fermentation in brine on olive fruits, with and without the addition of thyme, sage, and rosemary hydrosols (obtained by HD). The research focused on changes in oil content, bioactive components, antioxidant capacity, and sensory qualities. The findings revealed that the bioactive components in the hydrosols played a significant role during fermentation, leading to higher oil yields in olives fermented with hydrosols compared with those fermented in brine alone. Both hydrosol- and brine-fermented olives showed decreased levels of total phenols and flavonoids compared with fresh olives due to structural breakdown during fermentation. Although the antioxidant capacities were similar across all samples, olives fermented in thyme and rosemary hydrosols had the highest values. Grassino et al. [138] have highlighted the HD of green and roasted coffee, along with its byproducts, such as coffee silver skin and spent coffee grounds, as an effective method for isolating bioactive compounds. These compounds, known for their beneficial bioactivities, hold promise for use in functional beverages or to enrich other drinks. Lamine et al. [139] focused on utilising discarded citrus tree biowaste to recover bio-based bioactive molecules that are highly demanded at the industrial level. Their research demonstrated that the citrus HD bio-residue wastewater is a sustainable resource capable of producing plant complexes with significant bioactive potential, befitting food additives, nutritional supplements, functional foods, and beverages. Another work [140] aimed to evaluate the effects of osmotic dehydration, with and without prior antioxidant enrichment, using *R. damascena* wastewater byproducts. These studies revealed that such byproducts are excellent natural antioxidant sources, making them ideal for developing high-quality, value-added functional foods.

#### 4.1.4. Enzymatic Browning Prevention by Hydrosols

Enzymatic browning occurs in some minimally processed foods, such as peeled and washed vegetables and fruits, and is a common problem that impacts consumer acceptance. Thus, searching for strategies to limit this phenomenon is an important research focus. A cheap, safe, and environmentally friendly approach to preventing enzymatic browning involves using natural compounds or extracts rich in bioactive compounds capable of inhibiting the enzymes leading to this process [141]. A review of the literature on using hydrosols as natural agents for reducing enzymatic browning is summarised in Table 5, with several studies highlighting their successful application in addressing this issue. Regarding HD byproducts, the study of Yu et al. [142] revealed that the primary components of *Cinnamomum cassia* and *Cymbopogon citratus* hydrosols (cinnamaldehyde and citral) provided positive effects on the tyrosinase (TYR) inhibition mechanism. This inhibitory activity is likely due to the carbonyl group’s ability to form a Schiff base, reacting through nucleophilic addition and dehydration condensation with the primary amino groups in the enzyme. Both cinnamaldehyde and citral were identified as non-competitive TYR inhibitors, with citral exhibiting a better affinity to TYR. The findings demonstrate that both hydrosols could effectively inhibit fruit and vegetable browning, making them suitable as anti-browning agents in food. SD byproducts were also reported to have this potential. In this context, Lante et al. [143] evaluated the anti-tyrosinase activity of three types of citrus hydrosols, focusing on TYR. The study found that all hydrosols inhibited commercial mushroom TYR at varied levels, depending on the substrate type and concentration. The anti-browning effect of these hydrosols was associated with their terpene components. These results show that hydrosols derived from discarded citrus peels could serve as natural TYR inhibitors in food, cosmetic, and medicinal products. Xiao et al. [144] explored the use of citronella and rose hydrosols to reduce browning in fresh-cut taro. The hydrosols effectively suppressed browning by decreasing the enzyme polyphenol oxidase (PPO) activity. Chemical analysis indicated that the hydrosols were rich in terpenoids, which likely contributed to this inhibitory effect. The study suggests that hydrosols hold promise as natural anti-browning agents for fresh-cut foods.

**Table 3:** Survey of different hydrosol applications as a natural sanitiser.

Plant Scientific Name	Extraction and Characterisation Methodologies	Main Results	Reference
*Salvia officinalis*, *Rosmarinus officinalis*, *Origanum onites* and *Thymus vulgaris*	Application: decontamination from fresh-cut parsley; Extraction procedure: HD; Chemical composition: not investigated; Biological characterisation: antimicrobial activity (decontamination assay).	Antimicrobial activity: after 60 min of treatment thyme, oregano, sage, and rosemary hydrosols caused 60.28%, 42.06%, 27.57% and 16.36% growth inhibition of *E. coli* O157:H7, respectively. While thyme and oregano hydrosols completely inhibited *S. aureus* growth, followed by 14.50% and 12.77% of inhibition from sage and rosemary hydrosols, respectively.	[145]
*Thymus vulgaris*, *Satureja hortensis*, *Rosmarinus officinalis*, *Salvia officinalis*, *Sideritis canariensis*, *Origanum onites* and *Laurus nobilis*	Application: decontamination of iceberg lettuce; Extraction procedure: HD; Chemical composition: GC–MS; Biological characterisation: antibacterial activity (decontamination assay).	Major compounds: rosemary hydrosol (1,8-cineole (49.90%)); bay leaf hydrosol (α-terpineol acetate (23.63%), 1,8-cineole (10.03%)); oregano hydrosol (thymol (51.18%), carvacrol (44.10%)); salvia hydrosol (*o*-cymene (10.26%)); summer savoury hydrosol (thymol (29.81%), carvacrol (19.12%), *o*-cymene (14.25%)); sideritis hydrosol (1,8-cineole (25.09%)), thyme (thymol (56.96%), *o*-cymene (13.45%)); Antibacterial activity: all studied hydrosols showed favourable results with a 3-log reduction in the pathogen number (of *E. coli* O157:H7, *L. monocytogenes* and *Salmonella*).	[130]
*Thymbra capitata*	Application: decontamination of surfaces as a natural sanitiser; Extraction procedure: not mentioned; Chemical composition: SPME + GC–MS; Biological characterisation: antibacterial activity (decontamination assay).	Major compounds: carvacrol (946.3 mg/L), 1-octen-3-ol (10.8 mg/L); Antibacterial activity: an approximated 5-log reduction of planktonic and biofilm of *Salmonella enterica* cells was obtained by applying 42% and 75% hydrosol solutions, respectively.	[128]
*Monarda citrodora*, *Monarda dydima*, *Monarda fistulosa*, *Grindela robusta*, *Origanum heracloticum*, *Satureja montana*, *Citrus aurantium*, *Cyanis segetum*, *Hamamelis virginiana*, *Melissa officinalis*, *Coridothymus capitatus*, *Mentha rotundifólia*, *Origanum vulgare*, *Rosmarinus officinalis* and *Salvia officinalis*	Application: safe cleaning of paper artwork; Extraction procedure: SD; Chemical composition: GC–MS; Biological characterisation: antifungal activity (broth microdilution assay).	Major compounds: *C. aurantium* hydrosol (linalool (47.70%), terpinolene (24.82%), α-terpineol (13.83%)), *M. fistulosa* hydrosol (carvacrol (49.68%), thymol (45.23%)); Antifungal activity: M. didyma*, M. fistulosa*, C. capitatus*, H.virginiana*, C. segetum*, C. aurantium subsp. Amara*, M. citrodora* hydrosols had fungicidal action (on *A. sydowi*, *C. spherospermum* and *P. chrysogenum*) at least at a dilution of 1:2.	[146]
*Mentha piperita*	Application: decontamination of shredded carrots; Extraction procedure: HD; Chemical composition: not investigated; Biological characterisation: antioxidant activity (ABTS assay, total phenolic content), antimicrobial activity (broth microdilution assay).	Antioxidant activity: IC_50_ value of 9.41 μL for mint hydrosol for ABTS assay; Antimicrobial activity: the washing treatments resulted in a significant decrease of the microbial growth of *E. coli* and *L. monocytogenes* (with a 0.57 10-log reduction).	[129]
*Origanum majorana*	Application: decontamination of shredded carrots; Extraction procedure: HD; Chemical composition: not investigated; Biological characterisation: antioxidant activity (DPPH and ABTS assays), antimicrobial activity (total viable count method).	General results: the combination of ascorbic acid with marjoram hydrosol increased carotenoid content and total soluble solids. The total viable counts of fungi were decreased by single or combined treatment (decreasing by 49.1% of fungi at the 9th day compared with the control) during storage.	[147]
*Citrus lemon* L. and *Mentha spicata* L.	Application: decontamination of salad vegetables; Extraction procedure: HD system; Chemical composition: not investigated; Biological characterisation: antimicrobial activity (plate count method).	Antimicrobial activity:*C. lemon* hydrosol decreased 7.38 log CFU/g in total microbial counts, and 7.35 log CFU/g on *E. coli* counts. *M. spicata* hydrosol reduced 6.99 log CFU/g in total microbial counts and showed no antimicrobial effect on *E. coli*.	[148]
*Coridothymus capitatus*, *Origanum hirtum*, *Rosmarinus officinalis*, *Salvia officinalis* and *Citrus aurantium*	Application: decontamination of food-contact surfaces (polystyrene and stainless steel); Extraction procedure: not mentioned (commercial samples); Chemical composition: not investigated; Biological characterisation: antimicrobial activity (broth microdilution method), antibiofilm activity.	Antimicrobial activity: MIC value of 125 μL/L on *L. monocytogenes* from C. aurantium* hydrosol; Biofilm formation inhibition: sub-MIC concentrations of C. aurantium* hydrosol (62.5 μL/L) inhibited biofilm formation in a value range of 20.6–38.2% (showing a low-to-moderate efficacy).	[149]
*Citrus limon*, *Thymus serpyllum* and *Thymus vulgaris*	Application: antiviral properties for reducing foodborne viral diseases; Extraction procedure: not mentioned (commercial samples); Chemical composition: SPME + GC–MS; Biological characterisation: cytotoxicity determination and virucidal effect.	Major compounds: *C. limon* hydrosol (limonene (53.45%), β-pinene (20.60%) and γ-terpinene (14.03%)), *T. serpyllum* hydrosol (thymol (84.01%)), *T. vulgaris* hydrosol (carvacrol (58.67%), linalool (17.11%), cymene (11.23%)); Virucidal effect: *T. vulgaris* and *T. serpyllum* hydrosols showed greater virucidal activity against MNV-1, by reducing in 2 log after treatment, *C. limon* reduced 1 log, both immediately and after 24 h of treatment.	[150]

HD = hydro distillation system, O157:H7 = serotype of the bacterial species *E. coli*; GC–MS = gas chromatography–mass spectrometry; SD = steam distillation system, SPME = solid-phase microextraction; ABTS = free radical scavenging assay; IC_50_ = concentration needed to inhibit 50% of oxidant species; DPPH = free radical scavenging assay; CFU = colony forming unit; MIC = minimal inhibitory concentration; MNV-1 = murine norovirus; * indicates the plant hydrosols with the most promising results.

**Table 4:** Survey of different hydrosol applications for food enrichment.

Plant Scientific Name	Extraction and Characterisation Methodologies	Main Results	Reference
*Cymbopogon citratus* and *Murraya koenigii*	Application: as natural flavouring in herbal ice cream; Extraction procedure: SD; Chemical composition: not investigated; Biological characterisation: citral content, nutritional composition, and sensory analysis.	General results: *C. citratus* hydrosol was identified as a good source of citral content. Regarding ice cream nutritional composition, protein and carbohydrate levels were similar for ice creams made with both hydrosols. However, ice cream made with *M. koenigii* hydrosol had higher fat and total solids content. Both ice creams received high sensory scores (colour and appearance, flavour, body and texture, and melting quality), with the *C. citratus* formulation being the most promising due to bioactive citral compounds offering health benefits.	[151]
*Rosa damascena* Mill.	Application: development of alginate beads with rose hydrosol for future food applications; Extraction procedure: not mentioned (commercial sample); Chemical composition: GC–MS; Biological characterisation: antioxidant activity (total phenolic and flavonoid content, DPPH, ABTS and CUPRAC assays).	Major compounds: phenethyl alcohol (65.34%), 3,7-dimethyl-1,7-octanediol (13.25%) and α-terpineol (10.20%); Antioxidant activity: 15% inhibition by DPPH assay, 33% inhibition by ABTS assay, and the highest antioxidant power of 270 μM/mL by CUPRAC assay.	[152]
*Cymbopogon citratus*	Application: incorporation into beverages; Extraction procedure: HD; Chemical composition: HS-SPME/GC–MS; Biological characterisation: microbial analyses of Matcha tea beverages (method adopted by Uprel, Lda, Portugal, counting total mesophiles).	Major compounds: geranial (213.79 mg/g) and neral (135.00 mg/g); Microbial analyses of Matcha tea beverages: the incorporation of hydrosols inhibited the proliferation of fungi after 79 days of storage, displaying an antifungal activity. Incorporation as a food ingredient resulted in great acceptance for the readily prepared beverage from a consumer perspective. Percentages higher than 40% (*v/v*) were negatively rated due to strong citrus flavour.	[153]

SD = steam distillation system; GC–MS = gas chromatography–mass spectrometry; DPPH = free radical scavenging assay; ABTS = free radical scavenging assay; CUPRAC = cupric reducing antioxidant capacity; HD = hydro distillation system; HS-SPME = headspace solid-phase microextraction.

**Table 5:** Survey of different hydrosol applications as a natural reducing enzymatic browning agent.

Plant Scientific Name	Extraction and Characterisation Methodologies	Main Results	Reference
*Citrus medica*, *Citrus lemon* and *Citrus sinensis*	Application: hydrosols as anti-browning agents for commercial mushrooms; Extraction procedure: SD; Chemical composition: GC–MS; Biological characterisation: tyrosinase inhibition activity, determination of quinone inhibition.	Major compounds: citron hydrosol (α-terpineol (16.81%), geraniol (15.35%) and citral (17.4%)), lemon hydrosol (α-terpineol (29.98%), geraniol (48.27%), and citral (28.84%)), and orange hydrosol (terpinolene (12.41%)); Tyrosinase inhibition: IC_50_ values of 5.9 μg for orange hydrosol, of 18.04 μg for citron hydrosol, and 38.57 μg for lemon hydrosol; Quinone inhibition: citrus hydrosols showed no inhibitory effect on quinone production.	[143]
*Cymbopogon nardus* and *Rosa* sp.	Application: hydrosols effect on enzymatic-browning of fresh-cut taro; Extraction procedure: SD; Chemical composition: GC–FID and GC–MS; Biological characterisation: colour measurement (browning index), total phenolic content, enzyme inhibition assay (PAL, POD, and PPO).	Major compounds: rosa hydrosol (2,3-dehydro-1,8-cineole (23.84%), 3-carene (21.26%), and 2-one-6-methyl-5-hepten (16.28%)), citronella hydrosol (citronellol (20.49%)); Browning index: treating fresh-cut taros with 100 and 500 mL/L with each hydrosol significantly reduced the browning index during 12 days of cold storage, compared with the control, as evidenced by colour measurements and visual inspection; Enzymes inhibition: rose and citronella hydrosols significantly reduced PAL, POD, and PPO, showing promise as an inhibitor for the enzymatic browning for foods.	[144]
*Origanum bilgeri*, *Origanum minutiflorum*, *Origanum vogelli*, *Origanum majorana*, *Origanum onites*, *Origanum syriaucm* and *Origanum vulgare*	Application: hydrosols as natural preservatives on PPO activity in fresh-cut mushroom; Extraction procedure: HD; Chemical composition: not investigated; Biological characterisation: enzyme inhibition assay (PPO).	Enzyme inhibition: *O. bilgeri* hydrosol provided 62.26% reduction of enzyme activity at the end of the 3rd storage day. Hydrosols administrations were found to be statistically significant when compared with control (*p* < 0.01) (enzyme inhibition on 1st storage day: *O. majorana*, *O. minutiflorum* and *O. vogelii*; on 3rd storage day: *O. bilgeri*, *O. syriacum* and *O. vogelli*).	[154]

SD = steam distillation system; GC–MS = gas chromatography–mass spectrometry; IC_50_ = concentration needed to inhibit 50% of enzyme activity; GC–FID = gas chromatography with diode array detection; PAL = phenylalanine ammonialyase; POD = peroxidase; PPO = polyphenol oxidase; HD = hydro distillation system.

### 4.2. Agricultural Applications

The global reliance on chemical pesticides, largely due to intensive agricultural practices, raises significant environmental and human health concerns. This has stimulated interest in finding natural components with anti-pest properties. Hydrosols provide an eco-friendly and straightforward alternative, as research has shown that these liquid byproducts of essential oil distillation have pesticidal properties. They can control harmful organisms affecting crops, including weeds, insects, nematodes, mites, bacteria, and fungi. A summary of these studies can be found in Table 6. The majority of the pesticidal activity was evaluated based on antifeedant effects against significant crop pests such as the Colorado potato beetle (*Leptinotarsa decemlineata* Say), cotton leafworm (*Spodoptera littoralis* Boisd), and green peach aphid (*Myzus persicae* Sulzer). The insecticidal properties of hydrosols were largely attributed to their phenolic compounds [155]. In this context, Petrakis et al. [156] examined the effects of hydrosols from *Origanum majorana*, *Mentha pulegium*, and *Melissa officinalis*, extracted using HD and microwave-assisted HD, on the survival of the aphid pest *Myzus persicae*. Over 24 h, hydrosols from *M. officinalis* and *M. pulegium* demonstrated the most significant inhibitory effect, whereas *O. majorana* hydrosols induced 10–15% aphid mortality. These results indicate that hydrosols hold considerable potential for pest control and may warrant further research. The insecticidal activity of SD hydrosols, e.g., *Eucalyptus* spp. hydrosols, were also investigated. According to Sharma and Kaur [157] and Dermane et al. [158], hydrosols from *Eucalyptus globulus* and *Eucalyptus camaldulensis* could be safe and environmentally friendly alternatives to chemical pesticides for pest control. Moreover, studies have highlighted the nematocidal properties of HD hydrosols from *Thymus citriodorus*, *Satureja hellenica*, and *Cuminum cyminum*, as well as SD *Allium sativum* hydrosols for managing root-knot nematodes such as *Meloidogyne incognita* and *Meloidogyne javanica*. These findings suggest that these hydrosols are promising eco-friendly nematocidal agents [159,160,161,162].

In recent studies, Rosińska et al. [163] investigated the potential of commercial *Origanum vulgare* and *Cocos nucifera* hydrosols against seed contamination by pathogenic fungi. Their findings indicate that *A. alternata*, *Cladosporium* spp. and *Fusarium* spp. can be significantly reduced or eliminated using both hydrosols at high concentrations. Another study [164] evaluated the effectiveness of *R. officinalis* HD hydrosol in suppressing the growth of seven fungal strains isolated from buckwheat grains. Fungal infections leading to seed deterioration are a significant issue today. The study found that hydrosols at concentrations as low as 5% effectively inhibited *Fusarium graminearum*. Depending on the species, a maximum hydrosol concentration of 15% reduced the growth of various *Fusarium* fungi by 35–80%. Since neither concentration of hydrosol impacted buckwheat grain germination, rosemary hydrosol may be a viable alternative for preventing fungal growth in grains. Politi et al. [16] tested lavandin HD hydrosols for their efficacy against the food insect pest *Tribolium confusum*. The hydrosols, obtained from the flowers and stems, demonstrated excellent repellent properties, with RD_50_ values (the repellent dose needed to achieve 50% repellence) of 3.6 µL/cm^2^ and 3.3 µL/cm^2^, respectively. The study also assessed the impact of lavandin hydrosols on the seed germination of *Raphanus sativus*, revealing that they could serve as a natural herbicide by reducing seed germination. Moreover, other studies [165,166,167,168,169], using both SD and HD distillation methodologies, have reported the great potential of hydrosols as bio-pesticides, highlighting the valorisation of these byproducts of EO extraction and promoting the circular economy.

**Table 6:** Survey of different hydrosols applications as biopesticides/fungicides.

Plant Scientific Name	Extraction and Characterisation Methodologies	Main Results	Reference
*Origanum majorana*, *Mentha pulegium* and *Melissa officinalis*	Extraction procedure: HD and microwave-assisted HD; Chemical composition: GC–FID and GC–MS; Biological characterisation: insecticidal and inhibition potential (on *Myzus persicae*).	Major compounds:*O. majorana* hydrosol (carvacrol (78.0%) and terpinen-4-ol (11.3%)), *M. pulegium* hydrosol (piperitone (97.9%)), and *M. officinalis* hydrosol (carvacrol (35.0%), neral (17.2%), and geranial (12.7%)); Insecticidal activity: *M. officinalis* and *M. pulegium* hydrosols had the strongest inhibitory effect, *O. majorana* hydrosol caused 10–15% aphid mortality after 24 h.	[156]
*Mentha pulegium* and *Mentha suaveolens*	Extraction procedure: SD; Chemical composition: GC–FID and GC–MS; Biological characterisation: insecticidal activity (on *Toxoptera aurantia*).	Major compounds: *M. suaveolens* hydrosol (piperitenone oxide (69.32%)), and *M. pulegium* hydrosol (carvacrol (39.37%), and piperitenone (10.05%)); Insecticidal activity: the mortality of black aphids treated by *M. suaveolens* and *M. pulegium* hydrosols reached 100% with 1 mL of concentration (LC_50_ = 0.01 mL), and 99% with 2 mL of concentration (LC_50_ = 0.5 mL), respectively.	[170]
*Daucus carota* subsp. *Sativus*	Extraction procedure: SD; Chemical composition: GC–MS; Biological characterisation: antifungal activity (agar disc diffusion method), conservation assay (protective and preventive activity).	Major compounds: myristicine (17.8%), (E)-methyl-iso-eugenol (16.6%), and methyl eugenol (11.9%); Antifungal activity: with 0.1 mL/L concentration, the hydrosol showed inhibitory effects of 89.9% on *P. expansum* and 86.5% on *B. cinerea*.	[171]
*Artemisia absinthium*	Extraction procedure: SD; Chemical composition: HPLC–MS; Biological characterisation: nematocidal activity (effect on juveniles, effect on egg hatching, effect on juvenile infection capacity).	Major compounds: (*5Z*)-2,6-dimethylocta-5,7-diene-2,3diol (31.2%); Nematocidal activity: hydrosol showed strong nematocidal effects on *M. javanica*, suppressing nematode egg hatching (>95%) after 5 days. In-vivo tests showed a reduction in the root penetration rate on tomato.	[172]
*Ocimum basilicum* and *Ruta chalepensis*	Extraction procedure: HD; Chemical composition: GC–FID and GC–MS; Biological characterisation: insecticidal activity (on *Aphis gossypii* and *Tetranychus urticae*).	Major compounds:*O. basilicum* hydrosol (linalool (66.5%) and eugenol (18.9%)), *R. chalepensis* hydrosol (2-nonanone (77.0%)); Insecticidal activity: *O. basilicum* hydrosol (46% and 64% of mortality rate on *A. gossypii* and *T. urticae*, respectively), *R. chalepensis* hydrosol (50% and 56% of mortality rate on *A. gossypii* and *T. urticae*, respectively).	[22]
*Marrubium vulgare*	Extraction procedure: HD; Chemical composition: GC–MS; Biological characterisation: antifungal activity (radial growth technique), in-vivo antifungal assay (on *P. expansum*).	Major compounds: methyl eugenol (65.5%) and a α-bisabolol (12.5%); Antifungal activity: hydrosol showed percentages of inhibition of 85.6% for *B. cinerea*, 77.3% for *A. alternata* and 89.8% for *P. expansum*; In-vivo antifungal assay: 0.15 mL/L of hydrosol showed preventive and protective effects of 100% up to 20 days, and 80% up to the 25th day.	[173]
*Thymus citriodorus*	Extraction procedure: HD; Chemical composition: GC–MS; Biological characterisation: nematocidal activity (on *Meloidogyne incognita* and *Meloidogyne javanica*).	Major compounds: geraniol (44.06%); Nematocidal activity: EC_50_ values of 38.95 and 7.46% (*v/v*), for 1 and 2 days, respectively, for *M. incognita*. EC_50_ value of 4.35% (*v/v*) at the 2nd day for *M. javanica*.	[159]
*Satureja hellenica*	Extraction procedure: HD; Chemical composition: GC–MS; Biological characterisation: nematocidal activity (on *Meloidogyne incognita* and *Meloidogyne javanica*).	Major compounds: carvacrol (50.1%) and borneol (20.4%); Nematocidal activity: with dilution of 0.5 *v/v*, for both species, the percentage of dead second stage juveniles was more than 70% after 48 h, and more than 90% after 96 h immersion. No significant difference was observed for egg differentiation or for hatching inhibition using the hydrosol.	[161]
*Lavandula officinalis*, *Rosmarinus officinalis* and *Salvia officinalis*	Extraction procedure: SD; Chemical composition: not investigated; Biological characterisation: antioxidant activity (total phenolic and flavonoid content, TEAC and DPPH assays), antimicrobial activity (agar disc diffusion method).	Antioxidant activity: rosemary* and sage* hydrosols showed the strongest antioxidant potential for both assays; Antimicrobial activity: rosemary* hydrosol exerted activity on *S. aureus*.	[174]
*Lavandula x intermedia* Emeric ex Loisel	Extraction procedure: HD; Chemical composition: GC–MS and UHPLC–DAD–ESI–HR–MS, NMR spectroscopy; Biological characterisation: repellence activity (on *Tribolium confusum*), allelopathic activity.	Major compounds: flower hydrosol (linalool (43.8%), 1,8-cineole (25.4%) and camphor (12.8%)), stem hydrosol (linalool (34.4%), 1,8-cineole (28.9%) and camphor (15.4%)); Insect repellence bioassay: RD_50_ values of 3.58 and 3.26 μg/cm^2^ for flower and stem hydrosols, respectively; Allelopathic activity: seed germination was completely inhibited by flower hydrosols, while the stem hydrosols reduced the germination percentage by 24%.	[16]
*Cuminum cyminum*	Extraction procedure: HD; Chemical composition: GC–MS; Biological characterisation: nematocidal activity (on *Meloidogyne incognita* and *Meloidogyne javanica*).	Major compounds: γ-terpinen-7-al (42.9%), cumin aldehyde (31.5%) and α-terpinen-7-al (20.9%); Nematocidal activity: for both nematode species, an increase in the paralyzed second stage juvenile was observed with the increase in hydrosol concentration (5 to 50%) or exposure time. After immersion in a 50% hydrosol dilution, there was a noticeable decrease in egg differentiation.	[160]
*Origanum vulgare*, *Thymus vulgaris*, *Citrus lemon* and *Citrus sinensis*	Extraction procedure: SD; Chemical composition: not investigated; Biological characterisation: antifungal activity (agar dilution method), in-vivo antifungal assay (against *Botrytis cinerea*—grey mould).	Antifungal activity: IC_50_ values for growth inhibition 2.5% (*v/v*) of *O. vulgare* hydrosol, 2.1% (*v/v*) of *T. vulgaris* hydrosol, 0.7% (*v/v*) of *C. sinensis* hydrosol, and 7.6% (*v/v*) of *C. limon* hydrosol; In-vivo antifungal assay: hydrosols alone reduced the disease incidence percentage and disease severity index by 25% and 30%, respectively. While combinations of hydrosols and essential oils induced reductions of 70% and 76%, respectively.	[175]
*Salvia fruticose*, *Ocimum basilicum*, *Dracocephalum moldavica*, *Mentha spicata*, *Salvia officinalis*, *Melissa officinalis*, *Origannum onites* and *Thymus kotschyanus*	Extraction procedure: HD; Chemical composition: not investigated; Biological characterisation: allelopathy activity (on *Amaranthus retroflexus*)	Germination inhibition: considering the pure form of hydrosol (100%) there was a reduction in germination with M. spicata*, M. officinalis* and T. kotschyanus* hydrosols.	[176]
*Satureja montana* and *Citrus aurantium* var. *amara*	Extraction procedure: not mentioned (commercial sample); Chemical composition: provided by the producers; Biological characterisation: antimicrobial activity (micro and microdilution assays), disease incidence experiments.	Major compounds:*S. montana* hydrosol (carvacrol (87.79%) and thymol (13.88%)); *C. aurantium* hydrosol (linalool (47.70%), terpinolene (24.82%), α-terpineol (13.83%)); Antimicrobial activity: MIC values (% *v/v*) for *S. montana* hydrosol (25 on *E. amylovora*, 50 on *P. savastanoi*, 25 on *X. vesocatoria*, and 12.5 on *A. vitis*); for *C. aurantium* hydrosol (6.25 on *E. amylovora*, 1.6 on *P. savastanoi*, 3.1 on *X. vesocatoria*, and 0.8 on *A. vitis*); Disease incidence experiments: 4.5% *v/v* showed resistance reduction in 50% protection.	[177]
*Salvia officinalis*, *Rosmarinus officinalis* and *Lavandula angustifolia*	Extraction procedure: SD; Chemical composition: GC–MS and HPLC–DAD; Biological characterisation: antioxidant activity (total phenolic and flavonoid content, TEAC, CUPRAC, DPPH, superoxide anion scavenging and metal chelating assays), inhibition of acetylcholinesterase activity.	Major compounds: sage hydrosol (camphor (81.57%) and thujone (15.02%)); rosemary hydrosol (camphor (37.52%), verbenone (34.80%), and 1,8-cineole (15.42%)); lavender hydrosol (linalool (29.03%), coumarin (15.47%) and α-terpineol (14.21%)); Antioxidant activity: sage* hydrosol exhibited the highest activity (2.09 μM/g for TEAC, 4.33 μM/g for CUPRAC, 264.14 μg/mL for IC_50_ DPPH, 622.12 μg/mL for IC_50_ superoxide, 502.10 μg/mL for IC_50_ metal chelating); Inhibition of enzyme activity: sage* and rosemary* hydrosols inhibited the enzyme activity.	[178]
*Monarda didyma*	Extraction procedure: HD; Chemical composition: GC–MS; Biological characterisation: fumigant toxicity assay (on *Drosophila suzukii*), contact toxicity assay, survival assay, food intake assay, egg-laying assay.	Major compounds: carvacrol (59%) and thymol (38%); Toxicity assays: fumigant assay showed that hydrosols do not cause mortality at the tested concentrations. For the contact assay, hydrosol showed a LC_50_ of 5.03 μL/mL after 48 h; Food intake assay: hydrosol resulted in a significant decrease in total food intake; Egg-laying assay: hydrosol caused a significant reduction in the number off egg laid in two different oviposition assays.	[179]

HD = hydro distillation system; GC–FID = gas chromatography with diode array detection; GC–MS = gas chromatography–mass spectrometry; SD = steam distillation system; LC_50_ = lethal concentration for 50% of the nematodes; HPLC–MS = high-performance liquid chromatography–mass spectrometry; EC_50_ = concentration that caused 50% decrease in nematode; TEAC = Trolox equivalent antioxidant capacity; DPPH = free radical scavenging assay; UHPLC–DAD–ESI–HR–MS = ultra-high-performance liquid chromatography with diode array detection and high resolution electrospray ionization mass spectrometry; NMR = nuclear magnetic resonance; RD_50_ = concentration of the extract that repels 50% of the exposed insect; IC_50_ = concentration needed to inhibit 50% of the pathogen (for antifungal activity); MIC = minimal inhibitory concentration; CUPRAC = cupric reducing antioxidant capacity; IC_50_ = concentration needed to inhibit 50% of the oxidant species (for antioxidant activity); * indicates the plant hydrosols with the most promising results.

### 4.3. Pharmaceutical Applications

Hydrosols proved to be relevant options for pharmaceutical applications as they are valuable sources of biologically active compounds, acting as natural alternatives against antibiotic-resistant microorganisms. A summary of the most relevant studies devoted to the use of hydrosols in pharmaceutical applications is included in Table 7. Nazlic et al. [180] have identified the *Veronica* genus (family *Plantaginaceae*) HD hydrosol as rich in biologically active metabolites, demonstrating exceptional antioxidant potential for pharmaceutical applications. Erdoğan et al. [181] developed creams (oil-in-water emulsions) enriched with *Nigella sativa* hydrosol, reporting promising pharmaceutical effects, including excellent antioxidant activity, analgesic effect and wound healing potential. Concerning hydrosols produced by SD, Marino et al. [24] studied the effectiveness of *C. capitatus* hydrosol, both alone and in combination with conventional antimicrobial agents such as tetracycline and itraconazole. The study found that the hydrosol was effective against resistant bacteria and yeasts. This therapeutical combination could enhance antimicrobial efficacy and minimise side effects. The synergistic effects of hydrosol against resistant microorganisms suggest a promising approach, particularly when combined with conventional antibiotics, resulting in potential benefits for topical pharmaceutical formulations. Xin et al. [182] also reported hydrosols as potential antibacterial agents for pharmaceutical formulations, highlighting the notable antibacterial activity of the *Paeonia ostii* hydrosol against several common skin-infecting bacterial pathogens. Oliveira et al. [183] investigated the bioactivities of *Thymus mastichina* and *Cistus ladanifer* hydrosols when applied topically, including their antioxidant, anti-inflammatory, cytotoxic, wound-healing, and antimicrobial properties. Their results indicate that these hydrosols possess a biocompatible profile with significant anti-inflammatory effects, stressing their potential as active ingredients in pharmaceutical formulations to enhance skin health. In another work [15], commercial samples of *Lavandula angustifolia*, *Lavandula intermedia*, *Origanum hirtum*, *Satureja montana*, *Monarda didyma* and *Monarda fistulosa* hydrosols were studied. After performing their chemical characterisation and antimicrobial activity evaluation, it was concluded that hydrosol solutions with low concentrations of active ingredients showed excellent results in the treatment of skin infections by preventing antibiotic resistance, thus making these natural products relevant solutions for pharmaceutical companies who are looking for alternative antimicrobial solutions for topical applications. In the work of Mileva et al. [184], which focused on the determination of the chemical composition and pharmacological activities of *Rosa damascena* Mill., *Rosa alba* L., *Rosa centifolia* L. and *Rosa gallica* L., obtained according to different extraction methodologies, promising biological activities for the EOs and corresponding hydrosols were achieved, targeting application for natural healers.

### 4.4. Medicinal Applications

Relevant results concerning the use of hydrosols for medical applications have also been reported according to the literature summary provided in Table 8. For example, regarding hydrosol samples already on the market, Mutluay Yayla et al. [185] have investigated the preventive effect of the sage tea–thyme–peppermint hydrosol for oral rinse when used in combination with basic oral care procedures on chemotherapy-induced oral mucositis. The tests showed that oral mucositis did not occur in 70% of the intervention group patients, concluding that the hydrosol helped to maintain oral hygiene and pre-served the integrity of the oral mucosa. In the same area, Demirbolat et al. [186] studied the *Rosa damascena* Mill. hydrosol, with results indicating that the oral consumption of that hydrosol did not provide toxicity on haematology, renal, or on hepatic functions. Moreover, these hydrosols have also shown the potential to act as natural protective agents against diabetic cataracts.

Similarly, Hussein et al. [187] studied the chemical profile and anti-cancer potential of agarwood hydrosol obtained by HD, concluding that hydrosols possess anti-attachment and cytotoxic effects on Calu-3 lung cancer cells, which can be associated with compounds present in their composition, e.g., hentriacontanone, benzaldehyde and 1-triclosan. However, further studies are recommended to determine the IC_50_, the half-maximum inhibitory concentration values. Another study [188] identified the major components of ten *Paeonia x suffruticosa* hydrosols (obtained through HD) as geraniol, nerol, citronellol, geranic acid, phenylethyl alcohol, and 1,3,5-trimethoxybenzene. These components, primarily oxygenated compounds, could interact with the central nervous system by targeting three key antidepressant mechanisms: the sodium-dependent serotonin transporter, the 5-hydroxytryptamine receptor 1A, and monoamine oxidase type A. De Santis et al. [189] investigated the bioactivities and chemical profile of *Rubus idaeus* hydrosol obtained by SD, reporting significant cytotoxic activity against tumour cell lines (human adenocarcinoma and human acute promyelocytic leukaemia cells) and proliferation on health cells in addition to vigorous antioxidant activity. These properties make it a promising candidate for use in dietary supplements to promote human health.

### 4.5. Cosmetic Applications

The herbal and floral scents and the bioactive properties of hydrosols make them highly appealing as cosmetic ingredients. Their antibacterial and anti-inflammatory effects are beneficial for skin health. Additionally, hydrosols are favoured in skincare products due to their pH levels, which is similar to that of physiological skin, helping prevent dehydration and providing a refreshing effect. They are commonly used in products such as face and hair masks. Beyond cosmetology and aromatherapy, herb and plant extracts play crucial roles in supporting the immune system, defending against bacteria, and aiding in the regeneration of damaged tissues [190].

Table 9 provides a summary of hydrosol applications in cosmetic products. Considering HD bio-residues, and according to Prusinowska et al. [191], *Lavandula angustifolia* hydrosols are well-suited for creating various ingredients for diverse cosmetic applications. They could be ideal for the natural or organic cosmetics market. Kunicka-Styczyńska et al. [192] evaluated the preservative activity of lavender hydrosols in moisturising body gels, concluding that this byproduct is adequate to replace the aqueous phase, contributing to the maintenance of the formulation’s microbiological stability. Another study [193] has reported *Humulus lupulus* hydrosol to be a promising bio-active source for cosmetic formulations due to its antioxidant potential and anti-inflammatory efficacy. Yang et al. [194] investigated the effect of high-pressure homogenisation on rose commercial hydrosols to enhance their antioxidant capacity and stability. Higher pressures effectively stabilised bioactive compounds, boosting the hydrosols’ antioxidant ability. Increased pressures produce droplets with smaller sizes and higher surface charges, maintaining hydrosols’ physical stability and enabling long-term storage, which is beneficial for cosmetical formulations.

Regarding hydrosols produced by SD, Smiljanić et al. [195] investigated the bioactivities and safety of hydrosols from nine Lamiaceae medicinal plants, reporting an antioxidant capacity strongly correlated with their total phenolic content. In addition, all hydrosols appeared to be safe for prolonged skin exposure using controlled concentrations (lower than 2.5% in cosmetic formulations). The study of Tavares et al. [196] focused on hydrosols derived from forest waste, namely *C. lusitanica* and *C. ladanifer*, obtained by HD and SD, respectively, and reported the presence of hydrosols with a composition rich in oxygen-containing monoterpenes, justifying their anti-inflammatory power. These properties make them suitable for the perfumery and/or the cosmetic industry. Panwar et al. [197] studied citrus hydrosols as flavouring and odour agents. The authors pointed out a wide range of cosmetic applications through the development of lotions, bath soaps, and body sprayers. Moreover, hydrosols can be re-extracted to obtain pure antioxidants or employed as an ingredient to boost a product’s antioxidant activity without further purification [198].

**Table 7:** Survey of different hydrosol applications for pharmaceutical purposes.

Plant Scientific Name	Extraction and Characterisation Methodologies	Main Results	Reference
*Monarda citriodora*	Application: various uses as antimicrobial agent against pathogens involved in human infections; Extraction procedure: HD; Chemical composition: GC–MS; Biological characterisation: antimicrobial activity (broth microdilution test).	Major compounds: thymol (66.4%) and carvacrol (28.6%); Antimicrobial activity: MIC values (% *v/v*) of 12.5–25% on *E. faecalis*, 25% on *E. faecium*, 12.5–25% on *E. coli*, 25% on *S. pyogenes*, 25% on *K. pneumoniae*, 12.5–25% on *S. aureus*, 50% on *P. aeruginosa*, 25% on *C. albicans*, 12.5% on *C. glabrata*, 12.5% on *C. parapsilosis*, 50% on *C. tropicalis*, 12.5% on *M. caribicca*, 12.5% on *S. cerevisiae*, 50% on *A. sydowii*, 50% on *C. cladosporioides*, 50% on *P. chrysogenum*, and 25% on *S. chartarum.*	[199]
*Oliveria decumbens*	Application: emulsion formulation to prevent oxidative stress and related diseases; Extraction procedure: HD; Chemical composition: GC–MS; Biological characterisation: antioxidant activity (ABTS, DPPH, H_2_O_2_ scavenging, hydroxyl radical scavenging, superoxide radical scavenging, nitric oxide scavenging, nitrite scavenging, linoleic acid oxidation inhibition, low density LDL oxidation inhibition, and TBARS assays).	Major compounds: carvacrol (52.94%) and thymol (37.63%); Antioxidant activity: IC_50_ values (μg/mL) of emulsions of EO + hydrosol at a concentration range of 20–200 μg/mL: 28 for ABTS, 44 for DPPH, 226 for H_2_O_2_, 92 for hydroxyl radical, 104 for superoxide ion, 116 for nitric oxide, 75 for nitrite, 192 for linoleic acid oxide, 130 for LDL, and 122 for TBARS.	[200]
*Coridothymus capitatus* (L.) Reichenb. Fil.	Application: combination therapy between natural compounds and drugs for improving antimicrobial action and reducing side effects; Extraction procedure: SD; Chemical composition: GC–MS; Biological characterisation: antimicrobial activity (broth microdilution method).	Major compounds: carvacrol (93.11%); Antibacterial activity: the order of susceptibility to hydrosol (MIC values ranged from 12.5 to 50% *v/v*) was *B. subtilis* = *S. aureus* ATCC > *S. aureus* MRSA = *S. epidermidis* = *L. monocytogenes* > *P. aeruginosa* strains; Antifungal activity: the order of susceptibility to hydrosol (MIC values ranged from 6.25 to 50% *v/v*) was *C. glabrata* > *C. albicans* = *C. guilliermondii* = *C. parapsilosis* > *C. krusei* = *C. tropicalis* > *C. norvegensis* = *C. lusitaniae* = *C. valida* strains.	[24]
*Veronica saturejoides* Vis. Ssp. *saturejoides*	Application: various uses as antioxidant agent in pharmacy technology; Extraction procedure: HD; Chemical composition: GC–MS; Biological characterisation: antioxidant activity (total phenolic and flavonoid content, DPPH and ORAC assays).	Major compounds: Prenj hydrosols (trans-p-mentha-1(7),8-dien-2-ol (31.75%), (E)-caryophyllene (24.52%) and methyl eugenol (13.35%)) and Kamenisca hydrosols (*trans*-p-mentha-1(7),8-dien-2-ol (36.63%), (E)-caryophyllene (12.25%), *allo*-aromadendrene (11.53%) and methyl eugenol (11.92%)); Antioxidant activity: for DPPH assay (0.225 μmol/mL (34.84% inhibition) and 0.323 μmol/mL (49.26% inhibition), for Prenj and Kamenisca hydrosols, respectively), for ORAC assay (0.559 and 0.679 μmol/mL, for Prenj and Kamenisca hydrosols, respectively).	[180]
*Lavandula x intermedia*	Application: evaluation of the therapeutic benefits of antimicrobial nano-emulsion formulation; Extraction procedure: not mentioned (commercial sample); Chemical composition: GC–MS; Biological characterisation: antibacterial activity (microdilution method)	Major compounds: pure hydrosol (1,8-cineole (52.9%), camphor (19.6%) and linalool (12.6%)), hydrosol in nano-emulsion (1,8-cineole (18.6%), camphor (32.9%) and linalool (20.6%)); Antibacterial activity: MIC values (% *v/v*) of 0.75 on *E. coli* and 0.06 on *B. cereus* for nano-emulsion hydrosol.	[201]
*Lavandula angustifolia*, *Lavandula intermedia*, *Origanum hirtum*, *Satureja montana*, *Monarda dydima* and *Monarda fistulosa*	Application: topical application for healing skin infections; Extraction procedure: not mentioned (commercial sample); Chemical composition: GC–MS; Biological characterisation: antimicrobial activity (broth microdilution test) on *S. aureus* MRSA, *S. aureus* MSSA, *S. pyogenes*, *E. faecalis*, *E. faecalis* VRE, *Enterococcus faecium*, *C. albicans*, *C.parapsilosis*, *C. glabrata*, *C. tropicalis*; *T. soudanense*, *T. tonsurans*, *T. rubrum*, *T. violaceum* and *M. canis*.	Major compounds:*L. angustifolia* hydrosol (β-linalool (42.15%), terpinen-4-ol (20.23%) and α-terpineol (19.01%)), *L. intermedia* hydrosol (β-linalool (34.17%), camphor (22.12%), and 1,8-cineole (19.08%)), *O. hirtum* hydrosol (thymol (100%)), *S. montana* hydrosol (carvacrol (85.79%) and thymol (13.88%)), *M. didyma* hydrosol (carvacrol (48.44%) and thymol (34.03%)), and *M. fistulosa* hydrosol (carvacrol (84.68%)); Antimicrobial activity: *O. hirtum* and *M. didyma* hydrosols were more active than the others against bacteria, yeast and dermatophytes. A proportion of 50% (*v/v*) of *O. hirtum* hydrosol was able to inhibit all bacteria growth. A proportion of 50% (*v/v*) of *S. montana*, *O. hirtum* and *M.* didyma hydrosols had an inhibitory and cytocidal effect against most dermatophytes, 25% (*v/v*) of *M. fistulosa hydrosol* was able to inhibit all strains.	[15]
*Melaleuca alternifolia*	Application: natural antimicrobial agent in health care products; Extraction procedure: SD (commercial sample); Chemical composition: GC–FID and GC–MS; Biological characterisation: antibacterial activity (agar disc diffusion method).	Major compounds: terpinen-4-ol (624.2 ug/mL) and 2-endo-hydroxy-1,4-cineole (112.8 μg/mL); Antibacterial activity: inhibition zones diameter ranging from 14.3 to 34.6 mm, where *B. subtilis*, *C. albicans* and *M. luteus* were very sensitive.	[202]
*Hypericum perforatum* L. ssp*. Veronese* (Schrank) H. Lindb	Application: natural antidepressant as a source of volatiles with antiproliferative, antioxidant and antiphytoviral activities; Extraction procedure: HD; Chemical composition: GC–MS; Biological characterisation: antioxidant activity (ORAC and DPPH assays), cytotoxicity assay (on cancer cells), antiphytoviral activity (on *N. tabacum* L. cv. Samsun).	Major compounds: myrtenol (12.33%); Antioxidant activity: 240.34 μmol/L for ORAC assay, 11.88% for DPPH inhibition; Cytotoxic effect: good activity on all three cancer lines with IC_50_ values of 8.3% on Hela, 8.81% on HCT116 and 7.05% on U2OS; Antiphytoviral activity: compared with the control on the 3rd, 5th and 7th days post inoculation, the percentages of inhibition of local lesions on hydrosol-treated plants were 50.37, 36.19, and 39.87%, respectively.	[203]
*Cistus ladanifer*, *Helichrysum italicum*, *Thymbra capitata* and *Ocimum basilicum*	Application: evaluate the potential of hydrosols as pharmaceutical ingredients; Extraction procedure: *C. ladanifer* and *H. italicum* (SD—commercial sample) and *T. capitata* and *O. basilicum* (HD); Chemical composition: GC–MS; Biological characterisation: acute toxicity test (on *Daphnia magna*).	Major compounds: *C. ladanifer* hydrosol (4-hydroxy-3-methylacetophenone (21.6%), myrtenol (11.2%) and *p*-cymen-8-ol (10.7%)), *H. italicum* hydrosol (α-terpineol (30.5%), carvacrol (29.6%) and 1,8-cineole (15.4%)), and *T. capitata* hydrosol (carvacrol (98.1%)), *O. basilicum* hydrosol (4-hydroxy-3-methylacetophenone (52.5%) and linalool (38.3%)); Acute toxicity assay: none of the tested hydrosols caused observable acute effects on *D. magna* after 48 h of exposure up to the highest concentrations tested (*C. ladanifer* and *H. italicum* hydrosols = 2000 mg/L, *O. basilicum* hydrosol = 8000 mg/L, *T. capitata* hydrosol = 400 mg/L).	[204]
*Thymus x citriodorus* (Pers.) Schreb.	Application: evaluation of the potential of hydrosols as active pharmaceutical ingredients for skin applications validating its anti-acne activity (controlling acne related bacteria, modulate inflammation and oxidation); Extraction procedure: SD (commercial sample); Chemical composition: GC–FID; Biological characterisation: antimicrobial activity (microdilution method), anti-biofilm activity, anti-inflammatory activity (nitric oxide production), antioxidant activity (DPPH assay), acute toxicity test (on *Daphnia magna*).	Major compounds: 1,8-cineole (26.3%), linalool (24.3%) and geraniol (13.9%); Antimicrobial activity: MIC value of 50% (*v/v*) on *C. acnes*; Anti-biofilm activity: a significant reduction of biofilm adhesion (approximately 70%) was present at the MIC value. Hydrosol was also able to impair preformed biofilms with disruptions ranging from 40 to 55% at half MIC and MIC value; Anti-inflammatory activity: hydrosol was able to inhibit nitric oxide production in a dose-dependent manner (reducing it by 1.56–6.25%); Antioxidant activity: IC_50_ values of 20.08%; Acute toxicity test: hydrosol caused no observable effects after 48 h of exposure (at highest concentration 2000 mg/L).	[205]
*Salvia rosmarinus* Spenn. Syn. *Rosmarinus officinalis* L., *Salvia officinalis* L., *Cupressus sempervirens* L.	Application: evaluate the hydrosols phytochemicals with pharmacological potential formulations; Extraction procedure: HD (commercial sample); Chemical composition: HS-SPME, GC–MS and UHPLC–HR-MS, NMR spectroscopy; Biological characterisation: allelopathy test, brine shrimp lethality test (on *Artemia salina*), cytotoxicity assay (MTT cell viability), antifungal activity, antioxidant activity (inhibition of horseradish peroxidase)	Major compounds: rosemary hydrosols (1,8-cineole (47.1%)); sage hydrosol (1,8-cineole (42.9%), α-thujone (24.3%), and β-thujone (14.7%)); cypress hydrosol (terpinen-4-ol (44.5%), and α-terpinyl acetate (10.6%)); Toxicological impact: rosemary* hydrosol showed higher toxicity than sage and cypress, inhibiting the germination of the Canasta variety, and the highest potential (mean mortality 95%) on *A. salina*; Cytotoxic effects: rosemary* hydrosol appears to be the best recommendation at the neuronal level, being at the same time non-toxic (5–50 μL/mL) to hypothalamic cells and protective against hydrogen peroxide-induced toxicity; Antifungal activity: rosemary* and sage* hydrosols displayed the best antimycotic profile with MIC values in the range of 7.81–6.25 μL/mL; Antioxidant activity: for cypress hydrosol (25.94% inhibition), rosemary hydrosol (19.53% inhibition), and sage hydrosol (31.58% inhibition).	[206]
*Rubus idaeus*	Application: evaluate the potential of hydrosols as active pharmaceutical ingredients; Extraction procedure: SD; Chemical composition: GC–MS; Biological characterisation: antioxidant activity (DPPH, ABTS and FRAP assays), antibacterial activity (agar diffusion method), cytotoxicity assay (MTT assay).	Major compounds: 1,8-cineole (50.8%), 3-carene (16.3%), and 2-heptanol (10.3%); regarding hexanoic hydrosol extract, all the compounds were lower than 10%; Antioxidant activity: for DPPH assay (6746 μM/100 mL), for ABTS assay (13.57 μM/100 mL), and for FRAP assay (307.48 μM/100 mL); Antibacterial activity: inhibition zone diameters of 7.67 mm on *B. cereus*, and 12 mm on *A. bohemicus*; Cytotoxic effect: selective cytotoxicity towards cancer cells with dose dependency. In contrast, the healthy cells tested showed increased proliferation when treated with the hydrosol.	[189]
*Rosa damascena*, *Rosa alba*, *Rosa centifolia* and *Rosa gallica*	Application: evaluate the potential of hydrosols as antioxidants and anti-herpesvirus nutraceuticals; Extraction procedure: SD; Chemical composition: not investigated; Biological characterisation: antioxidant activity (DPPH, ABTS and superoxide (O^2-^) inhibition assays), antibacterial activity (broth microdilution method), dehydrogenase activity (MTT test); cytotoxicity assay, and antiviral activity.	Results: all studied hydrosols exerted significant antioxidant activity and good anti-herpes simplex virus type-1 activity while maintaining a good toxicological safety profile toward normal cell lines. Hydrosols had a weak antiproliferative effect on *S. aureus* and showed no activity on Gram-negative bacterial and fungal.	[207]
*Cinnamomum osmophloeum*	Application: prospects for development of an additive or ingredient for natural remedy for erectile dysfunction; Extraction procedure: SD; Chemical composition: GC–MS; Biological characterisation: PDE5, ACE, AChE, and ARG2 inhibition assays.	Major compounds: *trans*-Cinnamaldehyde (65.03%); Inhibition assay: *C. osmophloeum* hydrosol showed therapeutic potential due to its bioactive compounds. It affects erectile dysfunction by inhibiting several enzyme activities, suggesting its potential to influence erectile function through multiple physiological pathways.	[208]
*Helichrysum italicum*	Application: natural extract to support skin regeneration; Extraction procedure: SD; Chemical composition: not investigated; Biological characterisation: scratch assay, immunostaining, and gene expression analysis.	General results: The study confirmed the regenerative properties of *H. italicum* hydrosol, showing a higher collagen deposition in cells treated with hydrosol concentrations of 20 and 30% compared with untreated cells, promoting the use of natural alternatives as a safe skin treatment for wounds.	[209]

HD = hydro distillation system; GC–MS = gas chromatography–mass spectrometry; MIC = minimal inhibitory concentration; ABTS = free radical scavenging assay; DPPH = free radical scavenging assay; H_2_O_2_ = hydrogen peroxide; IC_50_= concentration needed to inhibit 50% of oxidant species (for antioxidant activity); LDL = lipoprotein; TBARS = thiobarbituric acid reactive substances; SD = steam distillation system; ATCC = American type culture collection; ORAC = oxygen radical absorbance capacity; MRSA= methicillin-resistant *Staphylococcus aureus*; MSSA = methicillin-susceptible *Staphylococcus aureus*; VRE = vancomycin-resistant enterococci; RAW 264.7 = mouse macrophage cell line; GC–FID = gas chromatography with diode array detection; HS-SPME= headspace solid phase microextraction; UHPLC–HR-MS = ultra-high-performance liquid chromatography–high resolution-mass spectrometry; NMR = nuclear magnetic resonance; MTT = thiazolyl blue tetrazolium bromide test; FRAP = ferric-reducing antioxidant power assay; PDE5 = phosphodiesterase type five; ACE = angiotensin-I converting enzyme; AChE = acetylcholinesterase; ARG2 = arginase type 2; * indicates the plant hydrosols with the most promising results.

**Table 8:** Survey of different hydrosol applications as a natural antimicrobial or anti-cancer agent for medical purposes.

Plant Scientific Name	Extraction and Characterisation Methodologies	Main Results	Reference
*Salvia officinalis*, *Thymus vulgaris* and *Mentha x piperita*	Application: hydrosol oral rinse combined with basic oral care; Extraction procedure: not mentioned (sample obtained from EO producing company); Chemical composition: GC; Biological characterisation: prevention of chemotherapy-induced oral mucositis.	Major compounds: 2-hexanone, eucalyptol, menthone, camphor, pulegone, menthol, α-terpineol, piperitenone, thymol and carvacrol (% not mentioned); Oral mucositis inhibition: the incidence of oral mucositis was significantly lower with the intervention with hydrosol group compared with the control group on day 5 due to its antimicrobial and antifungal properties.	[185]
*Rosa damascena* Mill.	Application: hydrosols on lens enzyme activities in cataract development, haematology, and clinical biochemistry parameters in diabetic induction; Extraction procedure: not mentioned (sample obtained from a local distillery); Chemical composition: GC; Biological characterisation: haematology, clinical biochemistry, lens enzymatic activity.	Major compounds: citronellol (19.20%), geraniol (13.20%), and 2-phenylethanol (35.98%); Results: hematologic, hepatic and renal functions were all improved when 1515 mg/L rose hydrosol was consumed. Hyperglycaemia was also reduced, as was the production of advanced glycation end products. Rose hydrosols had the potential to prevent diabetic cataracts by blocking a critical enzyme in the polyol pathway.	[186]
*Aquilaria malaccensis*	Application: anti-cancer potential of hydrosols; Extraction procedure: HD; Chemical composition: GC–MS; Biological characterisation: cytotoxicity assay.	Major compounds: 6-octadecenoic acid (32.18%) and n-hexadecanoic acid (24.92%); Cytotoxic effect: at a concentration of 50 μL/mL with 12 h of exposure, agarwood hydrosol showed 100% inhibition of cell attachment and 95.1% of cytotoxic effects against Calu-3.	[187]
*Clematis flammula*	Application: colon carcinoma suppression; Extraction procedure: not mentioned; Chemical composition: GC–MS; Biological characterisation: antioxidant activity (DPPH and FRAP assays), cytotoxicity assay (MTT assay).	Major compounds: pentane-3-methyl, pentane-2-methyl, sulphurous acid, hexyl pentyl ester, cyclopentane methyl, 1-butene-3,3-dimethyl, n-hexane, cyclohexane (% not mentioned); Antioxidant activity: hydrosol showed good antioxidant potential (antioxidant effect close to the standards); Cytotoxic effects: hydrosol acted as a tumour suppressor, being a good sensitizer of TRAIL-induced apoptosis and an anti-inflammatory agent, by reducing the damage effect of colon carcinoma in vivo.	[210]
*Melissa officinalis*, *Achillea teretifolia*, *Achillea aleppica*, *Origanum onites* and *Salvia fruticosa*	Application: cytotoxic effect on the colorectal cell line; Extraction procedure: not mentioned; Chemical composition: not investigated; Biological characterisation: cytotoxicity assay (MTT method).	Cytotoxic effects: IC_50_ dose values of 25% for O. onites* hydrosol, 25% for M. officinalis* hydrosol and 50% for S. fruticose* hydrosol after 48 h against colorectal cancer.	[211]
*Dittrichia viscosa* (L.) Greuter (Asteraceae)	Application: as a natural anticancer agent; Extraction procedure: HD; Chemical composition: GC–MS and HPLC; Biological characterisation: antimicrobial activity (broth microdilution method), cytotoxicity assay (on cancer cells), antiphytoviral activity (on tobacco mosaic virus)	Major compounds:*p*-menth-1-en-9-ol (29.93%), 1,8-cineole (18.55%), linalool (11.67%), *cis*-sabinene hydrate (10.97%) and α-muurolol (10.25%); phenolic compound 3,4-dihydroxybenzoic acid (62.24 mg/L); Antimicrobial activity: bacterial and fungal growth was not affected by 25% dilution of hydrosol; Cytotoxic effects: significant inhibition of the division of cancer cells with IC_50_ values of 21.70% on Hela, 37.73% on HCT116, and 54.51% on U2OS; Antiphytoviral activity: compared with the control on the 3rd and 7th days post inoculation, the percentages of inhibition of local lesions on hydrosol-treated plants were 89.3 and 91.5%, respectively.	[212]
*Zanthoxylum schinifolium*	Application: as a therapeutic agent for allergic inflammatory diseases; Extraction procedure: SD; Chemical composition: GC–MS and HPLC; Biological characterisation: cytotoxicity assay (microculture tetrazolium assay), anti-inflammatory activity (reactive oxygen species generation and nitric oxide analysis).	Major compounds: estragole (50.86%) and camphor (30.68%); Cytotoxic effects: treatment with hydrosol (at concentrations of 25–100 ppm) for 24 h showed no significant cytotoxic effects; Anti-allergic effect: An amount of 25–75 ppm hydrosol reduced allergic symptoms. An amount of 100 ppm hydrosol inhibited β-hexominidase release, showing promising anti-allergic quality; Anti-inflammatory activity: An amount of 50–100 ppm hydrosol inhibited reactive oxygen species, and 25 ppm hydrosol significantly decreased nitric oxide production.	[213]
*Mentha pulegium*, *Mentha suaveolens* and *Mentha spicata*	Application: as natural antimicrobial agent on pathogenic bacteria; Extraction procedure: HD; Chemical composition: not investigated; Biological characterisation: antimicrobial activity (agar disc diffusion and macro-dilution methods).	Antimicrobial activity: the most sensitive bacteria was *S. aureus*, where *M. pulegium* hydrosol presented the highest potential (inhibition zone = 35 mm and MIC = 256 μL/mL), followed by *M. spicata* hydrosol (inhibition zone = 30 mm and MIC = 256 μL/mL), and *M. suaveolens* hydrosol (inhibition zone = 10 mm and MIC = 512 μL/mL).	[214]
*Mentha rotundifolia* and *Salvia officinalis*	Application: hydrosols on aging-related comorbidities; Extraction procedure: HD; Chemical composition: GC–MS; Biological characterisation: blood–brain barrier and gastro-intestinal absorption of compounds, antioxidant activity (DPPH and ABTS assays), physical change analysis, behavioural tests, tail immersion test for hyperalgesia, acetone test for cold allodynia, biochemical analysis.	Major compounds: *M. rotundifolia* hydrosol (1,8-cineole (34.45%), (E)-thujone (26.97%), and camphor (20.57%)), *S. officinalis* hydrosol (1,8-cineole (24.73%), thujone (17.98%), D-camphor (14.16%)); Antioxidant activity: *M. rotundifolia* (IC_50_ values of 95.54 and 101.01 μg/mL for DPPH and ABTS, respectively), *S. officinalis* hydrosol (IC_50_ values of 84.19 and 82.32 μg/mL for DPPH and ABTS, respectively); Behaviour changes: both hydrosol treatments significantly improved aging-related issues, including locomotion and motor coordination impairments, performance metrics, anxiety symptoms, and short-term spatial memory; Hyperalgesia and cold allodynia: both hydrosols reduce pain sensitivity in rats and show potential in improving cold allodynia in aging rats;	[215]
*Rosa centifolia* and *Rosa gallica*	Application: the potential use of hydrosols to protect the genome against damage caused by alkylating genotoxins; Extraction procedure: HD; Chemical composition: GC–MS; Biological characterisation: cytotoxic and genotoxic activity (on human lymphocytes).	Major compounds: *R. centifolia* hydrosol (phenylethanol (36.61%), citronellol + nerol (16.25%), and geraniol (17.55%)), *R. gallica* hydrosol (phenyethanol (42.47%) and geraniol (24.42%)); Cytotoxicity and genotoxicity: combined treatment with the two hydrosols showed strong anti-cytotoxic and anti-genotoxic effects against MNNG. Both rose products significantly reduced chromosome and micronuclei aberrations, demonstrating similar genoprotective potential when non-toxic concentrations were applied before MNNG exposure.	[216]

EO = essential oil; GC = gas chromatography; HD = hydro distillation system; GC–MS = gas chromatography–mass spectrometry; DPPH = free radical scavenging assay; ABTS = free radical scavenging assay; O157:H7 = serotype of the bacterial strain *E. coli*; TBARS = thiobarbituric acid reactive substances; Calu-3 = adenocarcinoma lung cancer cells; FRAP = ferric-reducing antioxidant power assay; MTT = thiazolyl blue tetrazolium bromide test; TRAIL= tumour necrosis factor-related apoptosis-inducing ligand; H_2_O_2_ = hydrogen peroxide; IC_50_ = concentration needed to inhibit 50% of oxidant species (for antioxidant activity); IC_50_ = the concentration of 50% cellular cytotoxicity of extracts on cancer cells (for cytotoxicity assay); ORAC= oxygen radical absorbance capacity; Hela= cervical cancer cell line; HCT116 = human colon cancer cell line; U2OS = human osteosarcoma cell line; SD = steam distillation system; HPLC = high-performance liquid chromatography; ppm = parts per million; MIC = minimal inhibitory concentration; MNNG = N-methyl-N′-nitro-N-nitrosoguanidine; * indicates the plant hydrosols with the most promising results.

**Table 9:** Survey of different hydrosol applications as a natural cosmetic ingredient.

Plant Scientific Name	Extraction and Characterisation Methodologies	Main Results	Reference
*Lavandula angustifolia*	Application: water phase replacement in cosmetics (body gel) acting as a preservative agent; Extraction procedure: HD; Chemical composition: GC–MS; Biological characterisation: antimicrobial activity (liquid macro dilution method).	Major compounds: fresh herb hydrosol (linalool (53.0%)), dry herb hydrosol (linalool (48.0%)), fresh flower hydrosol (linalool (43.6%)), and dry flower hydrosol (linalool (39.2%)); Antimicrobial activity: the dry flower hydrosol incorporated in moisturizing body gel presented the highest antibacterial activity against *E. coli* (population extinction in less than 10 days) and *S. aureus* (population extinction in less than 15 days). Fungi *Candida* sp. and *A. niger* were extremely sensitive to the formulation with dry lavender flower hydrosol with population extinction after 2 days.	[192]
*Lavandula angustifolia*	Application: assess the potential of hydrosols in providing a base with bioactive properties for natural cosmetics; Extraction procedure: HD; Chemical composition: GC–MS; Biological characterisation: antioxidant activity (ORAC and DPPH assays), antimicrobial activity (dilution and plate count method).	Major compounds: all of the hydrosol variants presented linalool (24.2–39.2%), linalool oxide (18.2–25.0%) and borneol (5.8–14.3%); Antioxidant activity: Proportions of 3.6% for DPPH assay and 3.8% for ORAC assay; Antimicrobial activity: low activity (population decrease of 0.05% on *B. subtilis*, *S. aureus*, *E. coli*, *P. aeruginosa*, *Candida* sp., *A. niger*, *P. expansum*);	[191]
*Cupressus lusitanica* Mill. and *Cistus ladanifer* L.	Application: assess the potential of hydrosols to be used in co-formulations in the perfumery and cosmetic industries; Extraction procedure: HD and SD; Chemical composition: GC–MS; Biological characterisation: antioxidant activity (ABTS assay, xanthine oxidase inhibiting activity, chelating metal ions capacity), antimicrobial activity (agar disc diffusion method), anti-inflammatory activity (albumin denaturation assay).	Major compounds:*C. lusitanica* SD hydrosol (umbellulone (47.5–48.2%) and terpinen-4-ol (23.5–24.0%)), *C. lusitanica* HD hydrosol (terpinen-4-ol (21.0–31.4%) and *p*-cymen-8-ol (10.5–15.7%)); *C. ladanifer* SD hydrosol (2,6,6-trimethyl cyclohexanone (9.1–12.4%) and *trans*-pinocarveol (5.0–12.6%)), *C. ladanifer* HD hydrosol (*trans*-pinocarveol (7.8%) and verbenone (7.8%)); Antioxidant activity: *C. lusitanica* hydrosol (3.3% for ABTS assay, 7.5% for superoxide assay, 18.1% for xanthine assay and 33.0% for chelating assay, using 30, 60, 50 and 200 μL of hydrosol, respectively), *C. ladanifer* hydrosol (8.2% for ABTS assay, 14.3% for superoxide assay, 25.3% for xanthine assay and 24.1% for chelating assay, using 30, 60, 50 and 200 μL of hydrosol, respectively); Antimicrobial activity: hydrosols showed no antimicrobial effect; Anti-inflammatory activity: 1 mL of both hydrosols showed 94–95% of inhibition.	[196]
*Myrica gale* L.	Application: assess the hydrosol potential to be a natural cosmetic ingredient; Extraction procedure: SD; Chemical composition: GC–FID and GC–MS; Biological characterisation: antimicrobial activity (broth microdilution method).	Major compounds:*M. gale* leaf hydrosol (1,8-cineole (28.6%), α-terpineol (15.6%) and terpinen-4-ol (14.3%)), *M. gale* flower hydrosol (1,8-cineole (44.2%), terpinen-4-ol (13.4%) and α-terpineol (11.3%)); Antimicrobial activity: 75% hydrosol concentration was able to inhibit the growth of 21% of *S. aureus*, 66% of *E. faecalis*, 8% of *E. coli*, 63% of *P. aeruginosa.*	[217]
*Lavandula angustifolia*, *Rosa damascena*, *Chamomilla recutita*, *Melissa officinalis*, *Mentha piperita*, *Juniperus communis*, *Melaleuca alternifolia*, *Hamamelis virginiana*, *Camellia sinensis*, *Tilia cordata*, *Prickly pear*, *Tilia platyphyllos*, *Rosmarinus officinalis* and *Anthemis nobilis*	Application: characterise the antioxidant capacity of commercial hydrosols available on the cosmetics market in Poland; Extraction procedure: not mentioned (commercial samples); Chemical composition: not investigated; Biological characterisation: antioxidant activity (total polyphenolic content, DPPH and FRAP assays).	Antioxidant activity: the percentage of DPPH radical scavenging amounted to 4.43–39.87%; redox potential was varied in the range of 1325.65–5794.38 μM/L, with the highest observed in the damascus rose* and green tea* hydrosols.	[218]
*Melissa officinalis*, *Daucus carotae*, *Thymus vulgaris*, *Lavandula officinalis*, *Hypossi officinalis* and *Chamomillae romanae*	Application: estimation of the potential topical application; Extraction procedure: HD; Chemical composition: not investigated; Biological characterisation: antioxidant activity (DPPH assay) and in-vivo safety and efficacy (on humans).	Antioxidant activity: only T. vulgaris* hydrosol achieved IC_50_, inhibiting 50–80% at a concentration of 10–60%; In-vivo safety and efficacy: the results show that hydrosols were safe for topical use (after 24 h of exposure) and could be beneficial to irritated skin (accelerating the pre-irritated skin recovery process).	[219]
*Piper nigrum*	Application: assess the potential of hydrosol to be used as a source of natural antioxidants for cosmetic formulations; Extraction procedure: not mentioned (commercial sample); Chemical composition: headspace-solid phase microextraction followed by GC–MS and GC–FID; Biological characterisation: antioxidant activity (DPPH assay).	Major compounds: α-terpineol (34.7%), borneol (17.3%), terpinen-4-ol (13.9%); Antioxidant activity: the DPPH neutralization was dependent on incubation time. The hydrosol presented higher antioxidant activity than the respective EO, presenting IC_50_ values of 4.450 and 0.993 mg/cm^3^ for no incubation and 20 min incubation, respectively.	[220]
*Origanum vulgare* spp. *hirtum*, *Salvia officinalis*, and *Mentha pulegium*	Application: assess the hydrosols potential to be implemented in the cosmetics industry as antimicrobial agents or preservatives, such as in the development of oral hygiene products; Extraction procedure: SD; Chemical composition: GC–MS; Biological characterisation: antimicrobial activity (M27-A2—reference method for broth dilution assay).	Major compounds: *O. vulgare* hydrosol (carvacrol (97.3%)), *S. officinalis* hydrosol (1,8-cineole (32.6%), α-thujone (22.4%), camphor (11.3%), borneol (22.6%)), *M. pulegium* hydrosol (pulegone (50.6%), piperitone (32.4%)); Antimicrobial activity: O. vulgare* hydrosol exerted antimicrobial activity against oral pathogens, presenting MIC values of 25 and 35% (*v/v*) on *Streptococcus mutans* (MBC value of 30% (*v/v*)) and *Candida albicans * (MFC value of 35% (*v/v*)), respectively.	[221]
*Juniperus phoenicea*	Application: assess the hydrosol’s potential as a natural antioxidant product; Extraction procedure: SD; Chemical composition: GC–MS and headspace GC–MS; Biological characterisation: antimicrobial activity (broth microdilution susceptibility testing), antioxidant and anti-inflammatory power (peripheral blood mononuclear cells viability and reactive oxygen species test).	Major compounds: 1,8-cineole (24.9%), camphor (13.0%), α-terpineol (38.1%); General results: The results indicate that hydrosol is a natural product capable of counteracting the pro-inflammatory and oxidizing environment typical of infectious skin diseases caused by *S. aureus*.	[222]

HD = hydro distillation system; GC–MS = gas chromatography–mass spectrometry; ORAC = oxygen radical absorbance capacity; DPPH = free radical scavenging assay; SD = steam distillation system; ABTS = free radical scavenging assay; GC–FID = gas chromatography with diode array detection; SD = steam distillation; HD = hydro distillation; FRAP = ferric-reducing antioxidant power assay; IC_50_ = concentration needed to inhibit 50% of oxidant species; * indicates the plant hydrosols with the most promising results.

## 5. Ecotoxicological Studies

EOs and plant extracts have long been used for their bioactive properties in response to the demand for natural products. However, despite the rising interest and anticipated increase in production, the potential environmental impacts have received relatively little attention [223]. Some studies have been carried out on the ecotoxicity effects of hydrosols on crustaceans (e.g., *Daphnia magna*), fishes, phytoplankton, and plants. In the work of da Silva et al. [224] and dos Santos Maia et al. [225] *Lippia alba* hydrosol showed a sedative and anaesthetic effect in juvenile *Colossoma macropomum* (freshwater fish) at 5% concentration, presenting a lethal concentration of 7.43%. Two other studies by Pino-Otín et al. [226,227] investigated the ecotoxicity of *Lavandula luisieri* and *Artemisia absinthium* hydrosols (acting as a biocide) on non-target soil organisms, demonstrating significant phytotoxic activity that inhibited root growth in *Allium cepa*, as well as suppressing the growth of a bacterial community from natural soil, thereby affecting their survival and metabolic functions. Using non-target organisms, Pino-Otín et al. [228] investigated the impact of citronellol (a compound found in some hydrosols) on rivers and soil. The results show that all three organisms (*D. magna*, *A. cepa*, and *Eisenia foetida*) were sensitive to the hydrosols, with a decrease in their metabolism. In more recent studies [229,230], the ecotoxicological effect of *Satureja montana* hydrosol showed that aquatic organisms (*D. magna* and *Vibrio fishery*) are sensitive to lethal concentrations < 1% hydrosol. Soil functions were impacted due to the strong bioactivity of the substances on earthworms. In the work of Ferraz et al. [204], the acute toxicity of *C. ladanifer*, *H. italicum*, *O. basilicum* and *T. capitata* hydrosols were studied, showing no toxicity to *D. magna*. Oliveira et al. [205], who investigated the *Thymus x citriodorus* hydrosol’s safety profile, concluded that that hydrosol caused no observable effects on *D. magna* after 48 h of exposure. The study by Politi et al. [206] reported that rosemary hydrosol exhibited potential toxicity (at 500 μL/mL) to *Artemia salina* and concluded that diluting the hydrosol could be adequate to ensure environmental safety.

Further research is also needed regarding the safety impact on potential consumers, to determine safe dosage levels for hydrosols, understand their variability, improve formulations, and ensure their efficacy. Hydrosols are particularly appealing because they lack many common side effects associated with essential oils, such as strong scents that can trigger headaches or cause skin irritation. When properly diluted, they can often be safely applied to the skin or ingested. However, depending on their intended use, hydrosols must meet certain criteria, including microbiological profile and volatile compound levels. Additionally, parameters such as aroma, appearance, composition, pH, storage conditions, and shelf-life stability must be clearly defined for successful commercialisation and safe application [14,42].

## 6. Conclusions and Future Perspectives

This work highlights the emerging significance of the byproducts of the essential oil industry, particularly hydrosols, as novel and promising sources of bioactive compounds with great potential for developing sustainable ingredients for different fields. These innovations offer significant advantages from a circular economy perspective by promoting the recovery and valorisation of products previously considered waste.

Although hydrosols remain a relatively underexplored topic, their remarkable potential as natural antimicrobials and antioxidants present excellent opportunities for novel applications. These include extending shelf life through functional packaging, serving as bioactive ingredients in cosmetic and pharmaceutical formulations, and acting as preservative and disinfection agents. Such applications could complete green production cycles and pave the way for creating new, nature-based products.

Recent advances have offered valuable insights into standardising extraction and characterisation methods, comparing laboratory and commercial samples, and combining hydrosols with other natural components to enhance their effectiveness. These developments are crucial for ensuring efficacy, quality control, and safety. Despite their promising potential, further research is required to establish safe concentration ranges and thoroughly evaluate their impact on final products, ensuring consumer safety and environmental sustainability. Ecotoxicology, a field that is gaining increasing attention, plays a crucial role in this effort.

In summary, hydrosols present an exciting platform for natural product development. Still, additional exploration is essential to unlock their full potential and establish safe and sustainable use across diverse applications.

## Figures and Tables

**Table 1:** A survey of studies on hydrosol composition detailing the tested plant matrix, extraction procedure and processing parameters, liquid–liquid extraction solvents, and identified major compounds.

Plant Scientific Name	Methodology	LLE Solvent	Major Compounds (≥10%)	References
*Ocimum basilicum* L.	Extraction procedure: HD; Parameters: 300/1.2 (g/L) for the fresh plant, 40/1.2 (g/L) for the dry plant, and 4 h distillation.	n-pentane	Fresh plant: methyleugenol (44.9%), linalool (10.3%) and eugenol (12.5%); dry plant: methyleugenol (45.8%).	[73]
*Rosmarinus officinalis* L.	Extraction procedure: HD; Parameters: 300/600 (g/g), distillation time not mentioned.	n-hexane	Camphor (24.9%), borneol (20.4%) and eucalyptol (19.8%).	[74]
*Eucalyptus alba*, *Eucalyptus camaldulensis* and *Eucalyptus tereticornis*	Extraction procedure: SD (Clevenger-type apparatus); Parameters: 100/1.5 (g/L), 2 and 4 h distillation.	n-hexane	*E. alba*: 1.8-cineole (39.1%), trans-pinocarveol (19.3%); *E. camaldulensis*: 1.8-cineole (52.6%); *E. tereticornis*: 1.8-cineole (30.7%).	[40]
*Citrus sinensis*, *Citrus reticulata*, *Citrus maxima* and *Citrus aurantifolia*	Extraction procedure: SD (Clevenger-type apparatus); Parameters: 100/1.5 (g/L), 90 min distillation.	n-hexane	*C. sinensis*: linalool (34.8%); *C. reticulata*: linalool (17.5%), α-terpineol (10.1%), trans-carveol (12.2%), citronellol (16.4%); *C. maxima*: trans-linalooloxide (21.3%), α-terpineol (13.0%), cis-linalool oxide (furanoid) (10.3%); *C. aurantifolia*: geranial (18.3%), nerol (15.8%), neral (15.3%), geraniol (13.1%), α-terpineol (14.6%).	[75]
*Picea mariana*	Extraction procedure: SD and HD (modified aluminium 20 L still); Parameters: HD: 200/15 (g/L); SD: 200 g (with 2 L/h of steam flow), 6 h distillation.	n-hexane	α-terpineol (29.3% SD and 33.5% HD).	[58]
*Mentha spicata*, *Zataria multiflora*, *Bunium persunicum* and *Trachyspermum ammi*	Extraction procedure: HD (Clevenger-type apparatus); Parameters: 200 g (volume not mentioned), 4 h distillation.	Petroleum ether	*M. spicata*: piperitone (38.27%), carvone (22.07%), pulegone (14.75%); *Z. multiflora*: carvacrol (55.94%), thymol (40.37%); *B. persunicum*: cuminol (32.48%), cuminic aldehyde (29.35%), γ-terpinen-7-al (19.41%); *T. ammi*: thymol (90.94%).	[41]
*Lindera umbrellata*	Extraction procedure: HD; Parameters: ratio plant/water not mentioned, 2 h distillation.	Not mentioned	Linalool (57.5%).	[76]
*Pinus cembra*	Extraction procedures: SD (medium scale copper alembic apparatus); Parameters: 12 kg (30 L of water for steam flow), 1 h distillation.	Hexane	α-terpineol (28–34%).	[77]
*Lavandula x intermedia*	Extraction procedure: SD (stainless-steel apparatus); Parameters: 250–300 g (steam flow not mentioned), 1 h distillation.	Ethyl acetate and n-hexane	linalool (35–40%); borneol (10–30%) and terpinen-4-ol (5–10%).	[78]
*Melaleuca alternifolia*	Extraction procedure: SD; Parameters: 500 g (steam flow not mentioned), 4 h distillation.	n-hexane	*trans*-caryophyllene (28.58%), terpinen-4-ol (16.27%), limonene (13.98%), and α-terpineol (10.10%).	[79]

LLE—liquid–liquid extraction; SD—steam distillation system; HD—hydro distillation system.
